# Systematics of ‘lithistid’ tetractinellid demosponges from the Tropical Western Atlantic—implications for phylodiversity and bathymetric distribution

**DOI:** 10.7717/peerj.10775

**Published:** 2021-04-02

**Authors:** Astrid Schuster, Shirley A. Pomponi, Andrzej Pisera, Paco Cárdenas, Michelle Kelly, Gert Wörheide, Dirk Erpenbeck

**Affiliations:** 1 Department of Earth and Environmental Sciences, Ludwig-Maximilians-Universität München, Munich, Germany; 2 Current affiliation: Department of Biology, Nordcee, Southern University of Denmark, Odense, Denmark; 3 Harbor Branch Oceanographic Institute, Florida Atlantic University, Ft Pierce, FL, USA; 4 Institute of Paleobiology, Polish Academy of Sciences, Warszawa, Poland; 5 Pharmacognosy, Department of Medicinal Chemistry, Uppsala University, Uppsala, Sweden; 6 National Centre for Coasts and Oceans, National Institute of Water and Atmospheric Research, Newmarket, Auckland, New Zealand; 7 GeoBio-Center, Ludwig-Maximilians-Universität München, Munich, Germany; 8 SNSB-Bayerische Staatssammlung für Paläontologie und Geologie, Munich, Germany

**Keywords:** Tetractinellida, Integrative taxonomy, Tropical Western Atlantic, Lithistiddemosponges

## Abstract

**Background:**

Among all present demosponges, lithistids represent a polyphyletic group with exceptionally well-preserved fossils dating back to the Cambrian. Knowledge of their recent diversity, particularly in the Tropical Western Atlantic Ocean (TWA) where they are common in deep waters, is scarce making any comparison between present and past major ‘lithistid’ faunas difficult. In addition, the lack of sufficient molecular and morphological data hamper any predictions on phylogenetic relationships or phylodiversity from this region. The Harbor Branch Oceanographic Institute (HBOI, Fort Pierce, Florida) holds the largest collection of TWA lithistid sponges worldwide, however, the majority remain to be taxonomically identified and revised.

**Principal Findings:**

In this study we provide sequences of 249 lithistid demosponges using two independent molecular markers (28S rDNA (C1-D2) and *cox1* mtDNA). In addition, a morphological documentation of 70 lithistid specimens is provided in the database of the Sponge Barcoding Project (SBP). This integrated dataset represents the largest and most comprehensive of the TWA lithistids to date. The phylogenetic diversity of ‘lithistid’ demosponges in the Bahamas and Jamaica are high in comparison to other TWA regions; Theonellidae and Corallistidae dominate the fauna, while Neopeltidae and Macandrewiidae are rare. A proposed tetractinellid suborder, one undescribed genus and several undescribed species are recognized and the Pacific ‘lithistid’ genera, *Herengeria* and *Awhiowhio*, are reported from the TWA for the first time. The higher-taxa relationships of desma-bearing tetractinellids are discussed and topics for revision suggested.

**Conclusion:**

This first integrative approach of TWA ‘lithistid’ demosponges contributes to a better understanding of their phylogenetic affinities, diversity and bathymetric distribution patterns within the TWA. As in the Pacific, the TWA ‘lithistid’ demosponges dominate deep-water habitats. Deeper taxonomic investigations will undoubtedly contribute to a better comparison between present major ‘lithistid’ faunas and their fossil record in the Mesozoic.

## Introduction

Among all present demosponges, lithistids represent a palaeontologically important polyphyletic group, with exceptionally well-preserved fossils dating back to the Cambrian (e.g., Pisera, 2002; Pisera, 2006), and several relict genera represented in living faunas today (e.g., Lévi, 1991; Pisera, 2002; Kelly, 2007; Kelly et al., 2003). Several key ‘lithistid’ demosponge faunas are relatively well known: ’lithistid’ demosponges are dominant components of seamount communities on the Norfolk Ridge and in the South-West Pacific (e.g., Lévi, 1991; Kelly, 2000; Kelly, 2007; Schlacher-Hoenlinger, Pisera & Hooper, 2005; Kelly et al., 2007), and their inventory, morphological identification and molecular systematics has been the focus of several studies (e.g., Schlacher-Hoenlinger, Pisera & Hooper, 2005; Schuster et al., 2015). Furthermore, large ‘lithistid’ assemblages are reported from continental shelves and caves of the North-East Atlantic (e.g., Carvalho, Pomponi & Xavier, 2015), and from seamounts in the Mediterranean (e.g., Maldonado et al., 2015).

However, the present-day lithistid species and their phylogenetic diversity in several marine bioregions including the Western Indian Ocean, Subantarctic regions including South Africa, Northern Pacific and Tropical Western Atlantic (TWA) are incompletely understood. While ‘lithistid’ demosponges in the TWA are reported from continental shelves, caves and slopes by Van Soest & Stentoft (1988), Reed & Pomponi (1997), and Pomponi et al. (2001), and many earlier reports of individual species (e.g., Sollas, 1888), the fauna is still poorly known with few descriptions and no molecular data. This greatly limits the understanding of their phylogenetic relationships, diversity and evolution.

Desma-bearing demosponges, historically referred to as ‘lithistid’ demosponges, form a polyphyletic group. Molecular systematics now group the majority of ‘lithistid’ demosponges (11 out of 13 families) to the order Tetractinellida Marshall, 1876. Eight of these families are assigned to the suborder Astrophorina Sollas, 1887 and three to the suborder Spirophorina *sensu* Morrow & Cárdenas, 2015 (Cárdenas et al., 2011; Morrow & Cárdenas, 2015; Schuster et al., 2015). Schuster et al. (2015) showed several ‘lithistid’ families such as Pleromidae Sollas, 1888, Desmanthidae Topsent, 1894 and Scleritodermidae (Sollas, 1888) to be polyphyletic, and Corallistidae (Sollas, 1888), Theonellidae Von Lendenfeld, 1903 and Phymatellidae Schrammen, 1910 to be monophyletic. However, the systematic affinities for families such as e.g., Siphonidiidae Von Lendenfeld, 1903, Azoricidae Sollas, 1888 and Neopeltidae Sollas, 1888, remain obscure due to few molecular data available. Hence, only 21 out of 40 ‘lithistid’ genera were evaluated in Schuster et al. (2015). The same study indicated that several spicule types convergently evolved within this sponge group. The families Scleritodermidae and Siphonidiidae were suggested to form a separate clade within Tetractinellida, but outside the two suborders Astrophorina and Spirophorina (Kelly Borges & Pomponi, 1994; Schuster et al., 2015). With the discovery and description of a new tetractinellid family Stupendidae (Kelly & Cárdenas, 2016), a sister group relationship of Stupendidae to a clade consisting of rhizomorine-desma-bearing Scleritodermidae, Siphonidiidae and Azoricidae Sollas, 1888 was recently indicated (Kelly & Cárdenas, 2016). However, understanding the higher taxonomic relationships within Tetractinellida including its lithistid lineages is still hindered by incomplete taxon sampling and sequencing of key taxa such as Thrombidae Sollas, 1888 or *Gastrophanella* Schmidt, 1879 (e.g., Kelly Borges & Pomponi, 1994; Cárdenas et al., 2011).

Aside from the report of ‘lithistids’ in some specific island regions of the TWA, such as Barbados (Van Soest & Stentoft, 1988), the Bahamas (Maldonado & Young, 1996; Reed & Pomponi, 1997), Cuba (Pisera, 1999), Dutch Antilles (Van Soest, Meesters & Becking, 2014), the deep Florida shelf (Pisera & Pomponi, 2015), and chemotaxonomic studies (Kelly Borges & Pomponi, 1994) the most comprehensive taxonomy based survey comprising nearly all island groups in the TWA was conducted by Pomponi et al. (2001). The main focus of a study of Pomponi et al. (2001) was the documentation of biodiversity and bathymetric distributions of ‘lithistids’, thus no morphological species descriptions, sequences or phylogenetic affinities of these specimens were included. Although Pomponi et al. (2001) concluded that ‘lithistids’ are an important and dominant group of deep, hard-bottom habitats in the TWA, no comprehensive integrative taxonomic approach using molecular and morphological data has yet been made to evaluate this large and unique collection of TWA ‘lithistid’ demosponges, which is to a large extent unidentified and awaits taxonomic revision. Their study was based on 36 expeditions and 450 submersible transects led by the Harbor Branch Oceanographic Institute (HBOI) from 1984 to 2000, and aimed to provide an inventory of the biodiversity and bathymetric distribution of TWA ‘lithistids’. As a result, 28 ‘lithistid’ species representing 18 genera and nine families were reported from the TWA. However, knowledge of the TWA ‘lithistid’ fauna still remains comparatively poorly known, but crucial for a better knowledge of their global diversity and their comparison to the Mesozoic ‘lithistids’.

**Figure 1:**
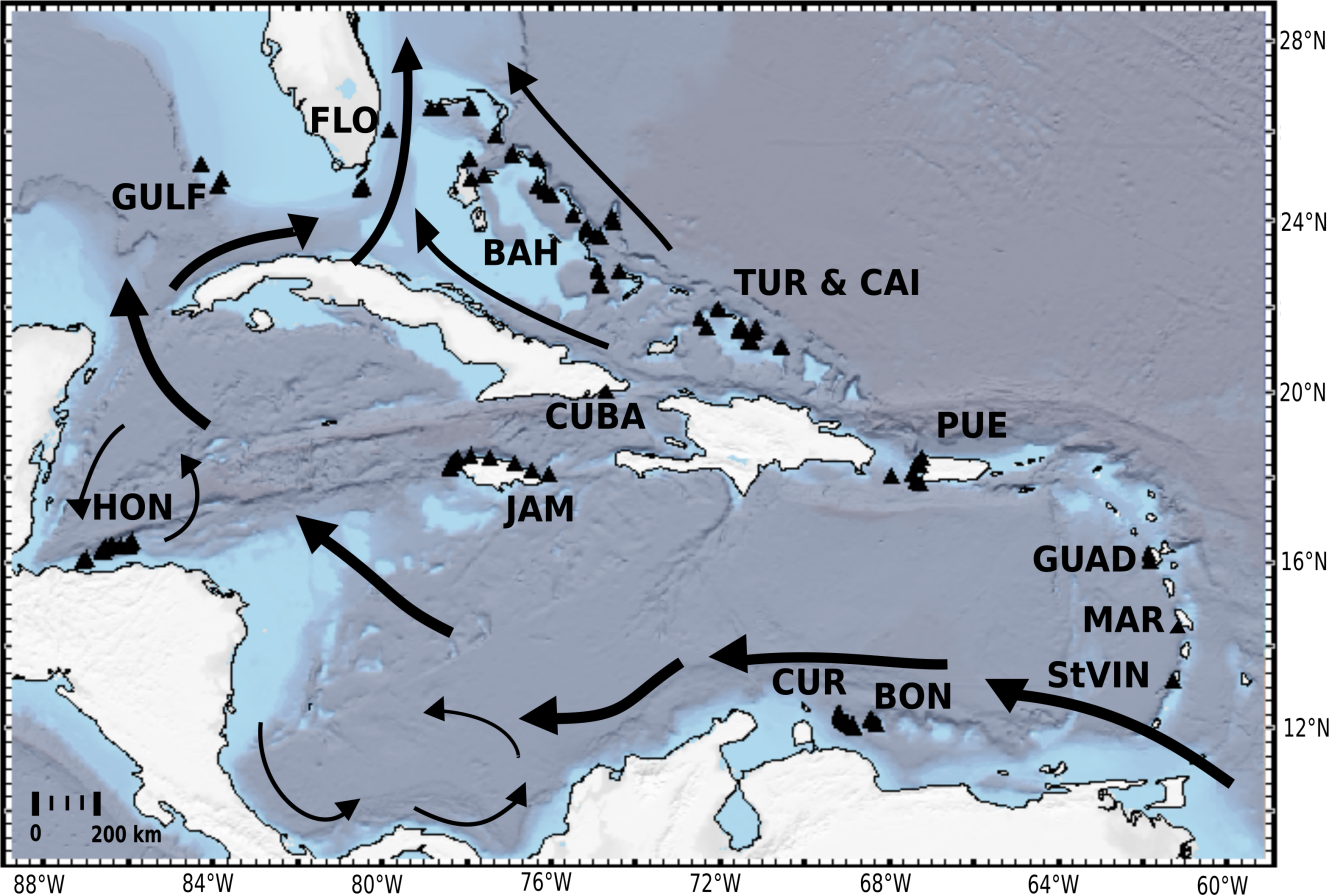
Distribution map of investigated HBOI and other desma-bearing tetractinellids and Vetulinidae from the TWA. Abbreviations correspond to the different locations (GULF, Gulf of Mexico; CUR, Curaçao; BON, Bonaire; StVIN, St. Vincent; MAR, Martinique; GUAD, Guadaloupe; PUE, Puerto Rico; JAM, Jamaica; HON, Honduras; TUR and CAI, Turks and Caicos; BAH, Bahamas; FLO, Florida). Arrows depict main surface currents. Map generated with GeoMapApp 3.6.3 (http://www.geomapapp.org, Ryan et al., 2009).

Therefore, the aim of this study is to (1) provide a general molecular phylogenetic overview of the subordinal classification within Tetractinellida; (2) focus on desma-bearing taxa from the TWA to provide a robust phylogeny for further analyses. In order to achieve this aim we investigated a large part of the extensive HBOI ‘lithistid’ collection (249 specimens) by means of generating independent molecular markers (*cox1* and 28S, C1-D2 region) from material collected between 1985 and 2011. Complementary to this we included *in situ* and SEM pictures of 71 taxa into the Sponge Barcoding Project (SBP). This study includes samples from almost all island groups in the TWA (Fig. 1) from depths ranging between 2 and 950 m, covering different geomorphological zonations as described in Reed & Pomponi (1997). The phylogenetic affinities of 31 out of the 35 ‘lithistid’ Tetractinellida genera are reconstructed.

## Materials and Methods

### Specimen collection and identification

Sponge samples were collected from the Tropical Western Atlantic (TWA) using the Johnson-Sea-Link manned submersibles with permission granted to Harbor Branch Oceanographic Institute by: the United States National Oceanic and Atmospheric Administration, National Marine Fisheries Service, Dry Tortugas National Park (letters of acknowledgement F/SER25:KM, F/SER23:PE, SER02-130) and the Florida Keys National Marine Sanctuary (permit numbers 2001-049, 2001-043); the Government of the Bahamas, Department of Fisheries; the Government of Honduras, Department of Fisheries (DIGEPESCA-638/97); the Government of Portugal, Parque Natural de Madeira; the Government of Jamaica, Jamaican Ministry of Foreign Affairs (diplomatic note no. 7/703/315); the Government of Bonaire, Bonaire Marine Park, and Netherlands Ministry of Foreign Affairs (note no. VADV-172/00); the Government of Panama (American Embassy Panama telegram no. 5570); the Puerto Rico Department of Natural And Environmental Resources; the Government of St. Vincent; the Government of France; and the Government of Turks and Caicos (American Embassy London telegram no. 12514). Sampling locations are indicated on the map (Fig. 1). These expeditions aimed to conduct a biodiversity inventory and collect samples for biomedical research focused particularly on sponges, octocorals and algae. Various habitats from the fore reef slopes and escarpments to the deep shelf slopes were sampled using either a claw, suction tube or scoop in depths from 0–1000 m. Sponge samples from this collection, were pre-identified by S.P. and M.K., and frozen and/or stored in 70% ethanol. For comparison, additional material from the Southwest Pacific (New Caledonia and New Zealand), and Indo-Pacific region, in the National Institute of Water and Atmospheric Research (NIWA) collection in Auckland and its invertebrate collection (NIWA Invertebrate Collection, NIC) in Wellington, New Zealand, were subsampled for molecular investigations. This material included subsamples of tetractinellids, which were collected by SCUBA diving during several expeditions across the Indo-Pacific and New Zealand, led by the Coral Reef Research Foundation (CRRF) under contract to the U.S. National Cancer Institute in Republic of Palau, identified by M.K. Six specimens (three *Geodia* spp. and three *Cinachyrella* spp.) from Jamaica and Norway were added from the Bavarian State Collection of Zoology (ZSM) in Munich, Germany (identification by Helmut Lehnert). Material from Marquesas were provided by Cécile Debitus (Institute de Recherche pour le Dévelopmement, IRD) sampled during the cruise DEBITUS Cécile (2009) BSMPF-1, RV Alis: 10.17600/9100030. More detailed information for all novel samples sequenced is provided in the Supplementary Material as well as in PANGAEA (http://www.pangaea.de) under the digital object identifier (DOI): 10.1594/PANGAEA.924148.

Undetermined samples from the TWA (all HBOI subsamples) were identified to the genus level according to their phylogenetic position relative to known species. Based on this, we selected 71 samples with distinct genotypes for a deeper morphological investigation. For those taxa we examined collection pictures, prepared thick sections and spicule and skeleton stubs for Scanning Electron Microscopy (SEM). We used the methodology outlined in Pisera & Pomponi (2015) to illustrate and evaluate morphological characters. Based on this, 249 specimens were identified to genus and/or species level. Morphological documentation for the 71 representative specimens are provided in the SBP (http://www.spongebarcoding.org/). SEM stubs and spicule slides including thick sections are deposited at the Bavarian State Collection for Paleontology and Geology (BSPG) Munich, Germany.

### Molecular investigations

Genomic DNA was isolated from small pieces of sponge tissue preserved in 70% ethanol using a modified protocol of the DNeasy (Qiagen) Blood and Tissue Kit, which included an additional centrifugation step just before transferring the lysate to the spin column. A Nano-Drop 1000 Spectrophotometer (Thermo Scientific) was used to quantify the isolated genomic DNA. Amplification of a fragment of the mitochondrial cytochrome c oxidase subunit 1 (*cox1*, partial ≈ 659 bp) was performed using the primers dgLCO1490 and dgHCO2198 (Meyer, Geller & Paulay, 2005). Additionally, a fragment of an unlinked nuclear ribosomal gene (28S; partition C1-D2, 768-832 bp) was amplified using the forward C1′ ASTR (Cárdenas et al., 2010) and the reverse universal D2 (Lê, Lecointre & Perasso, 1993) primers. Both amplifications follow the PCR protocol and settings outlined in Schuster et al. (2015). Amplification success was checked on a 1.5% agarose gel. For the majority of the 28S fragments we observed an additional non-specific shorter band at ≈ 650 bp, which was subsequently identified as originating from a bacterial template. Therefore, separation of double bands and PCR clean-up was performed using a modified freeze-squeeze method (Tautz & Renz, 1983), as described in Schuster et al. (2015). For sequencing of the 28S fragment, 6 µl of the remaining supernatant from the clean-up was used with the PCR primers and BigDye Terminator v3.1 (Applied Biosystems, Forster City, CA, USA) chemicals. For sequencing of *cox1* we used a 1:10 dilution of the PCR products together with the PCR primers and BigDye Terminator v3.1 chemicals. Sequencing was carried out on an ABI 3730 Genetic Analyzer at the Sequencing Service of the Department of Biology (LMU München). Sponge origin of novel sequences were tested by BLAST searches against NCBI GenBank (https://blast.ncbi.nlm.nih.gov/Blast.cgi). Raw trace files were post-processed by base-calling using CodonCode Aligner v.3.7.1.1 (CodonCode Corporation). Geneious v.8.1.8 (http://www.geneious.com, Kearse et al., 2012) was used for the assembly of forward and reverse reads. Sequences will be deposited at the European Nucleotide Archive (ENA) and the Sponge Barcoding Database (SBD) of the SBP under accession numbers SBD#1794 to SBD#2108.

### Phylogenetic reconstructions

Alignments were generated separately for *cox1* and 28S using MAFFT v.7 under the L-INS-I algorithm (Katoh & Standley, 2013) because of heterogeneous taxon sampling and moderate sequencing success of *cox1*. Saturation of both markers was evaluated using Xia’s test (Xia et al., 2003) as implemented in DAMBE v5.1.5 (Xia, 2013) which compares an estimated substitution saturation index (Iss) to a critical substitution saturation index (Iss.c). For the *cox1* dataset, sequences of *Halichondria panicea* (Pallas, 1766) (subclass: Heteroscleromorpha Cárdenas, Pérez & Boury-Esnault, 2012, order Suberitida Schmidt, 1870) and *Aplysina aerophoba* (Nardo, 1833) (subclass: Verongimorpha Erpenbeck et al., 2012, order Verongiida Bergquist, 1978) were chosen as outgroups. For the 28S dataset sequences of the order Sphaerocladina were chosen as outgroup. All outgroups have been used in earlier phylogenetic studies on tetractinellids (see e.g., Schuster et al., 2015; Kelly & Cárdenas, 2016). The final *cox1* alignment comprised 307 sequences of which 122 are newly generated sequences for this study. The alignment was 635 bp long, of which 295 bp were constant, 40 bp were parsimony uninformative and 300 bp were parsimony informative. The final 28S alignment comprised 474 sequences of which 305 are newly generated sequences for this study. In total this alignment was 905 bp long, of which 325 bp were constant, 66 bp parsimony uninformative and 514 bp parsimony informative. Both alignments from this study are freely available at OpenDataLMU 10.5282/ubm/data.221. Phylogenetic tree reconstructions for both datasets were performed on a parallel version of MrBayes v3.2.4 (Ronquist et al., 2012) on a Linux cluster. The most generalized GTR+G+I evolutionary model, indicated as the most suitable by jModelTest v.2.1.7 (Darriba et al., 2012), was used. Analyses were run in two concurrent runs of four Metropolis-coupled Markov-chains (MCMCMC) for 100,000,000 generations and stopped when the average standard deviation of split frequencies dropped below 0.01. The first 20% of the sampled trees were removed as Burn-in from further analyses.

### Inclusive molecular phylodiversity and abundance analyses

The Inclusive Phylogenetic Diversity (PD_I_) is the sum of all branch lengths of a gene tree connecting a set of taxa from the root of the tree to the tips of all phylogenetic branches spanned by this set of taxa (see e.g., Lewis & Lewis, 2005). To evaluate the PD_I_, a Maximum Likelihood (ML) tree was first calculated from the most comprehensive dataset (28S, C1-D2 partition) using RAxML 7.2.8 (Stamatakis, 2014). The GTRGAMMA nucleotide evolutionary model selected by jModelTest v.2.1.7 (Darriba et al., 2012) was taken with 1000 fast pseudo-replicated bootstraps. The resulting tree topology was used to calculate the PD_I_ for several areas in the TWA using a modified python script from Vargas et al. (2015). All non-TWA genera and all TWA genera less than five were excluded from this analysis. In total, the PD_I_ of Bonaire, Curaçao, Florida, Honduras, Jamaica, Puerto Rico and Turks and Caicos was calculated. In order to compensate for different sampling efforts across the seven regions, rarefaction curves (Sanders, 1968) were used for each location. The rarefaction curves were generated in RStudio (R Studio Team, 2014). Both scripts are available at https://bitbucket.org/molpalmuc/.

The relative abundance of eight ‘lithistid’ families from five depth zones (0–60 m; 61–150 m; 151–300 m; 301–600 m; 601–914 m) from the TWA was plotted and illustrated using ggplot2 (Wickham, 2009) as implemented in RStudio. These depth zonations follow Reed & Pomponi (1997) and Pomponi et al. (2001), which are based on the geomorphological observations of the sites sampled.

## Results and Discussion

### Integrative morphological and molecular systematics of ‘lithistid’ demosponges with focus on TWA species

#### Higher-taxa relationships of desma-bearing tetractinellids

**Figure 2:**
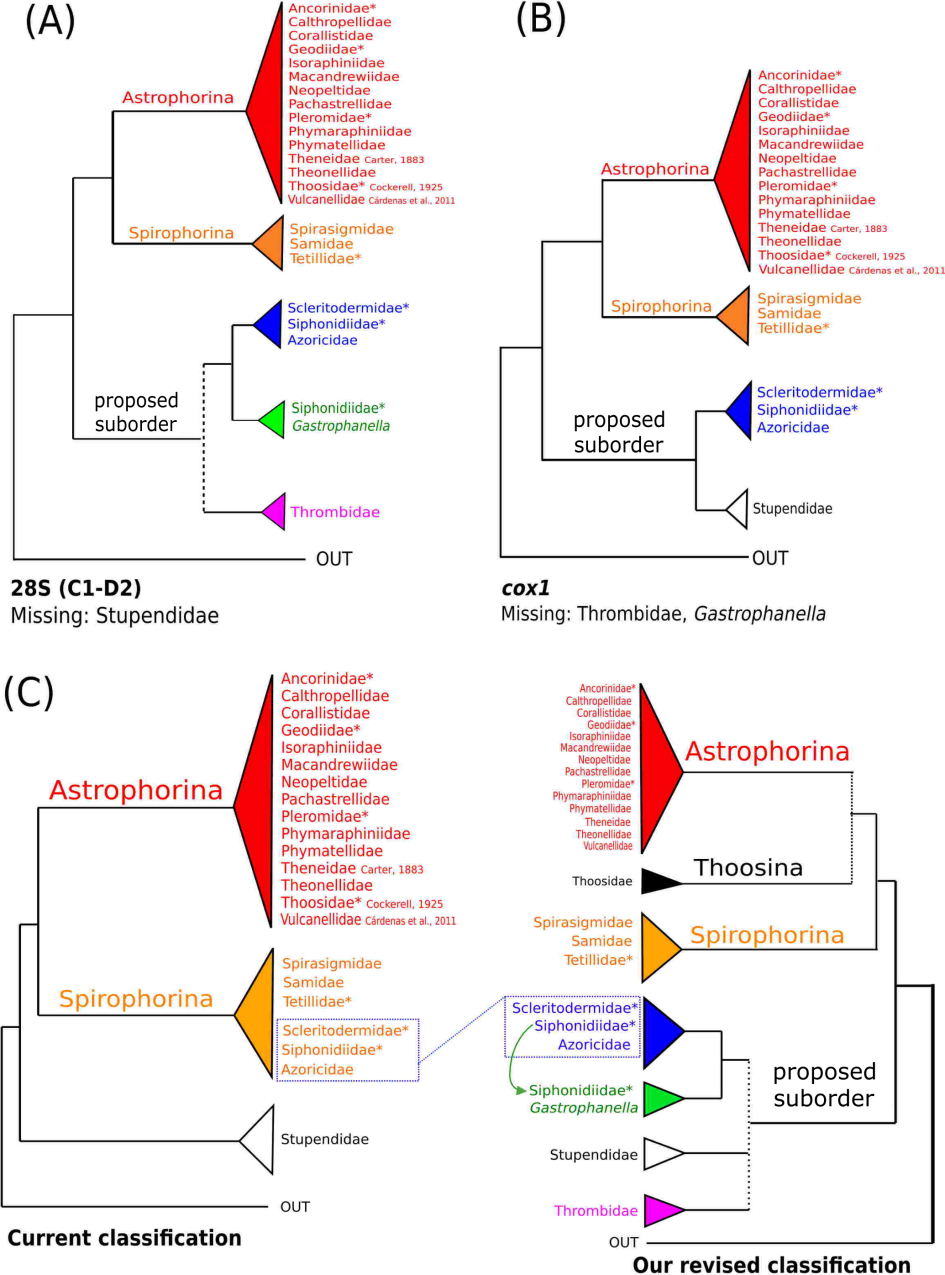
Schematic summary cladograms obtained from the 28S and *cox1* phylogenies indicating the higher-taxa relationships within the order Tetractinellida. (A) 28S (B) *cox1* summary tree with the suborders Astrophorina (red), Spirophorina (orange) and a proposed suborder (blue, green, pink and light gray) including all rhizoclone desma-bearing families and the families Thrombidae and Stupendidae. Stars behind family names indicate their proposed polyphyly. Dashed lines indicate the uncertainties of not supported topologies. (C) Comparison of current and revised classification including the proposed suborder Thoosina from Carballo et al. (2018).

**Figure 3:**
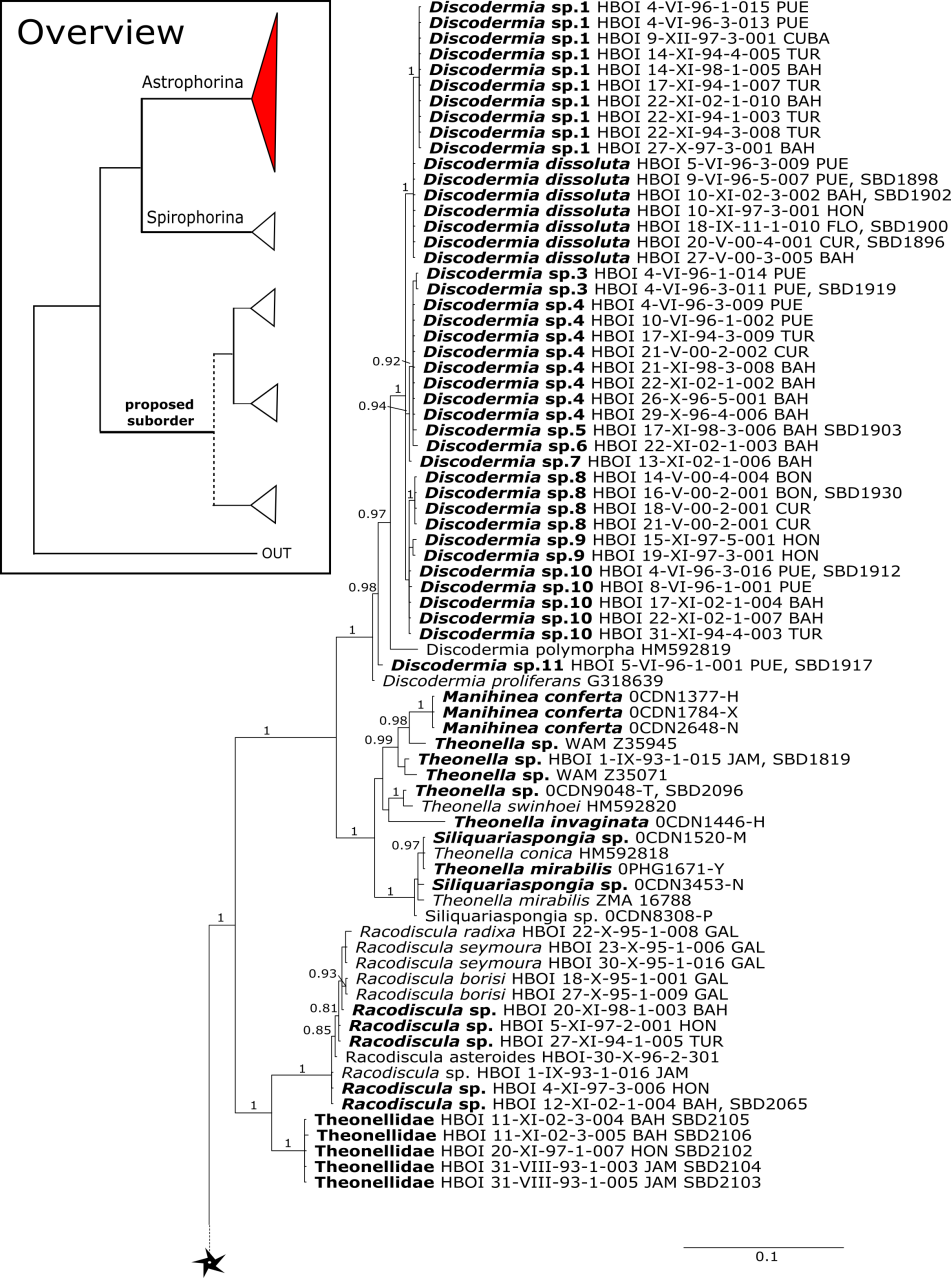
Bayesian Inference phylogeny of Tetractinellida based on 28S (C1-D2). Posterior probability (PP) values are provided above or below branches. Self-generated sequences are in bold. Numbers behind taxon names are either voucher numbers or GenBank/ENA accession numbers. Three letter code behind voucher numbers corresponds to the different locations (GULF, Gulf of Mexico; CUR, Curaçao; BON, Bonaire; GUAD, Guadaloupe; PUE, Puerto Rico; JAM, Jamaica; HON, Honduras; TUR, Turks & Caicos; BAH, Bahamas; FLO, Florida; GAL, Galápagos). Taxa where the morphology was investigated are indicated with their corresponding SBD#.

**Figure 4:**
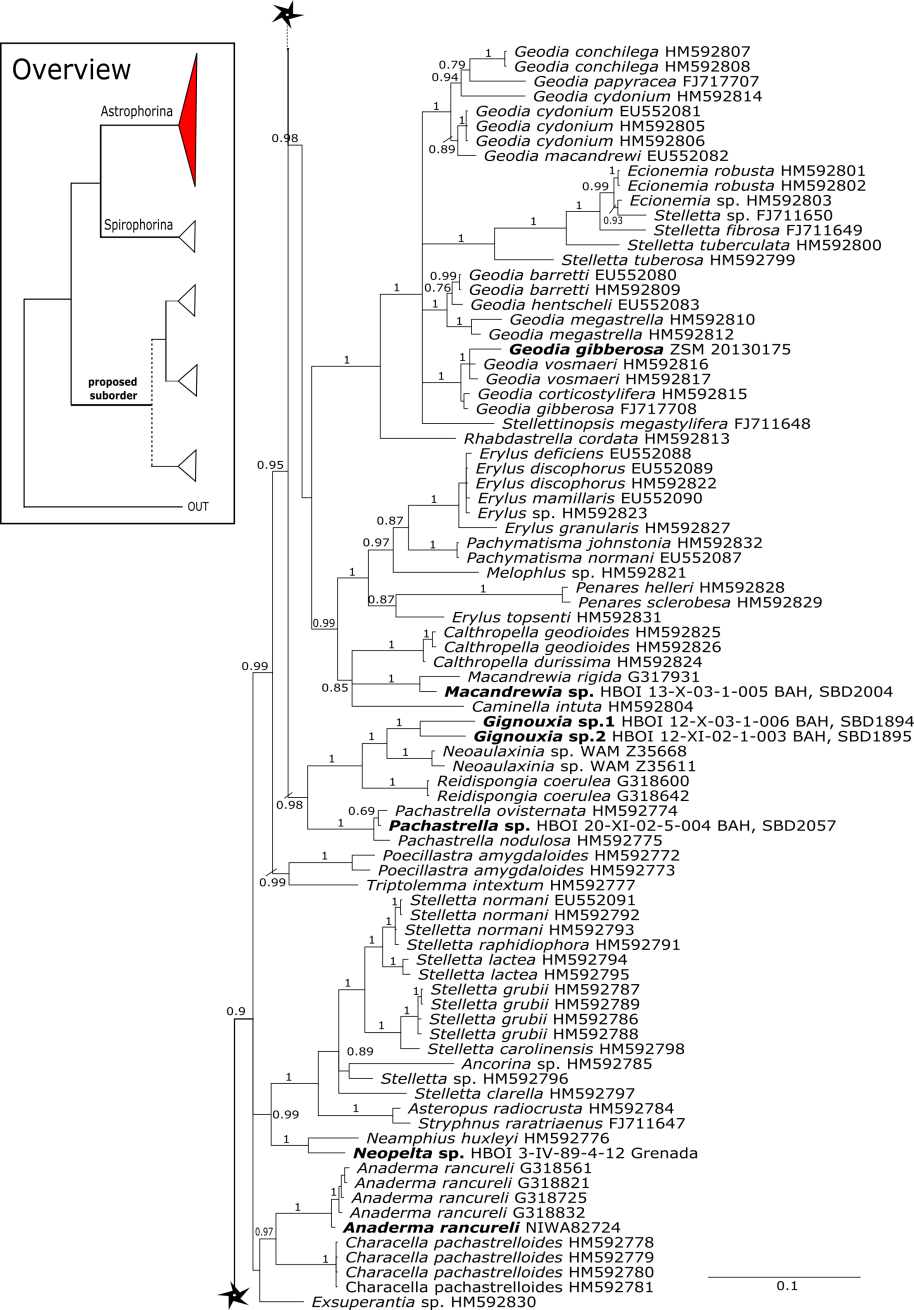
28S phylogeny continued. See caption in Fig. 3.

**Figure 5:**
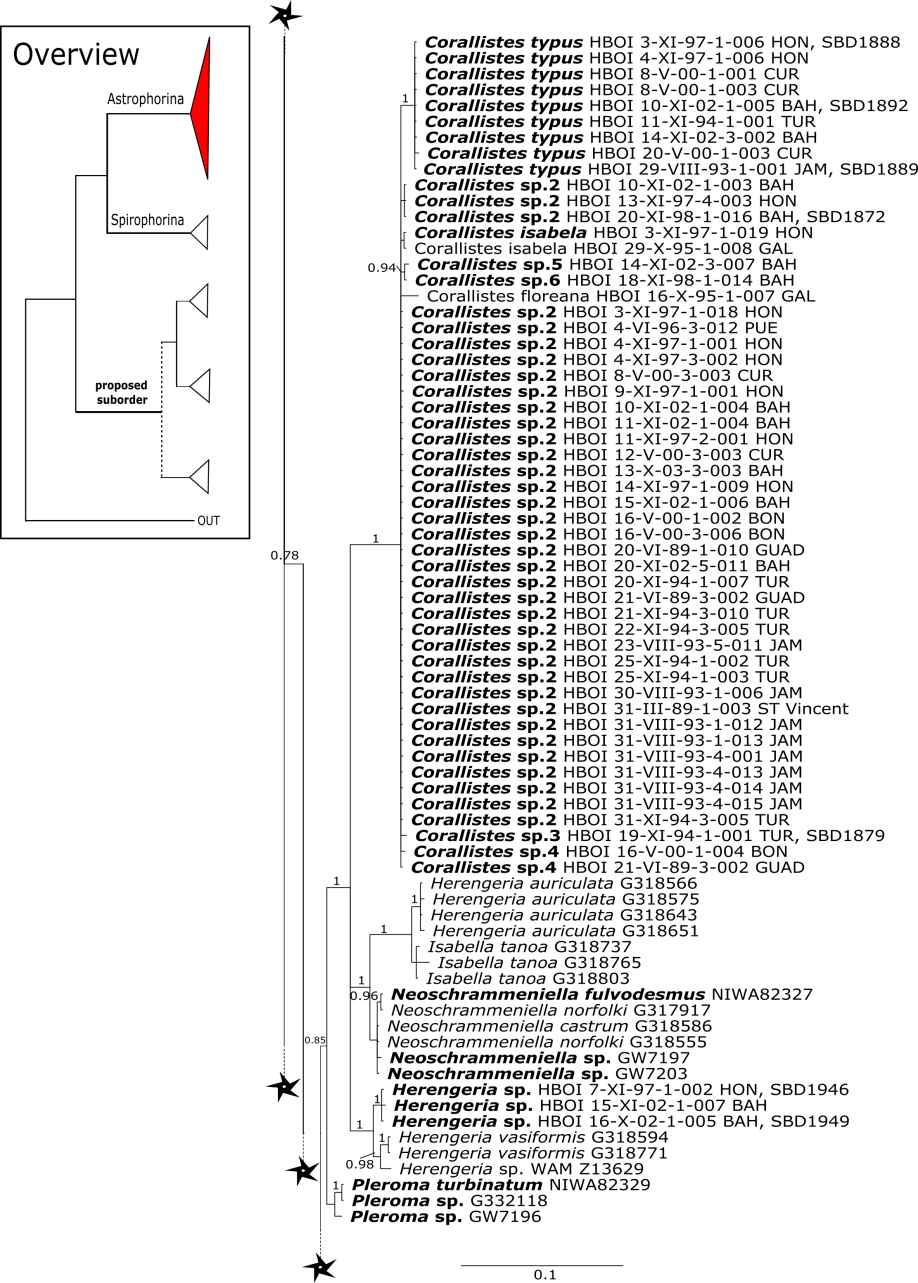
28S phylogeny continued. See caption in Fig. 3.

**Figure 6:**
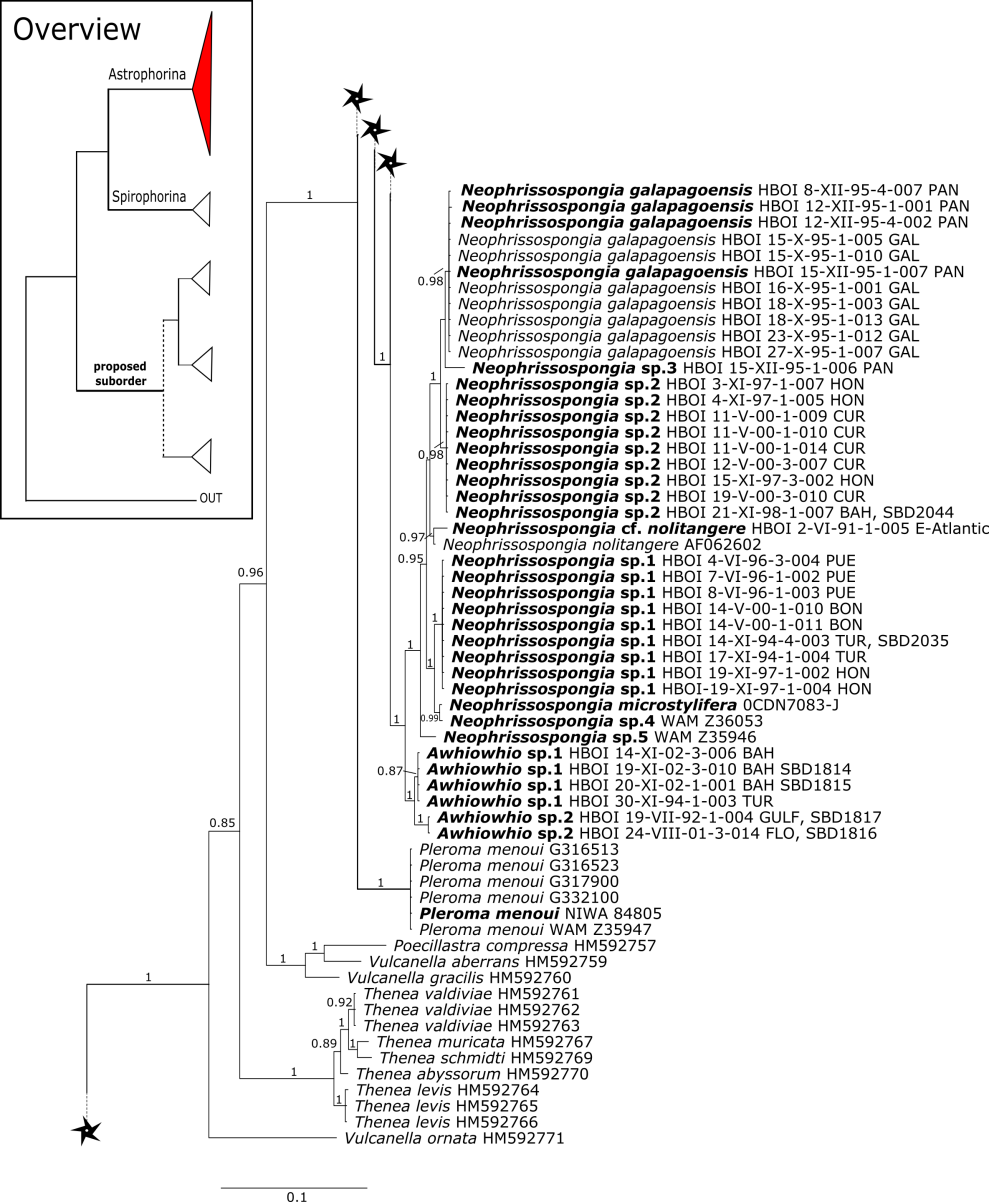
28S phylogeny continued. See caption in Fig. 3.

**Figure 7:**
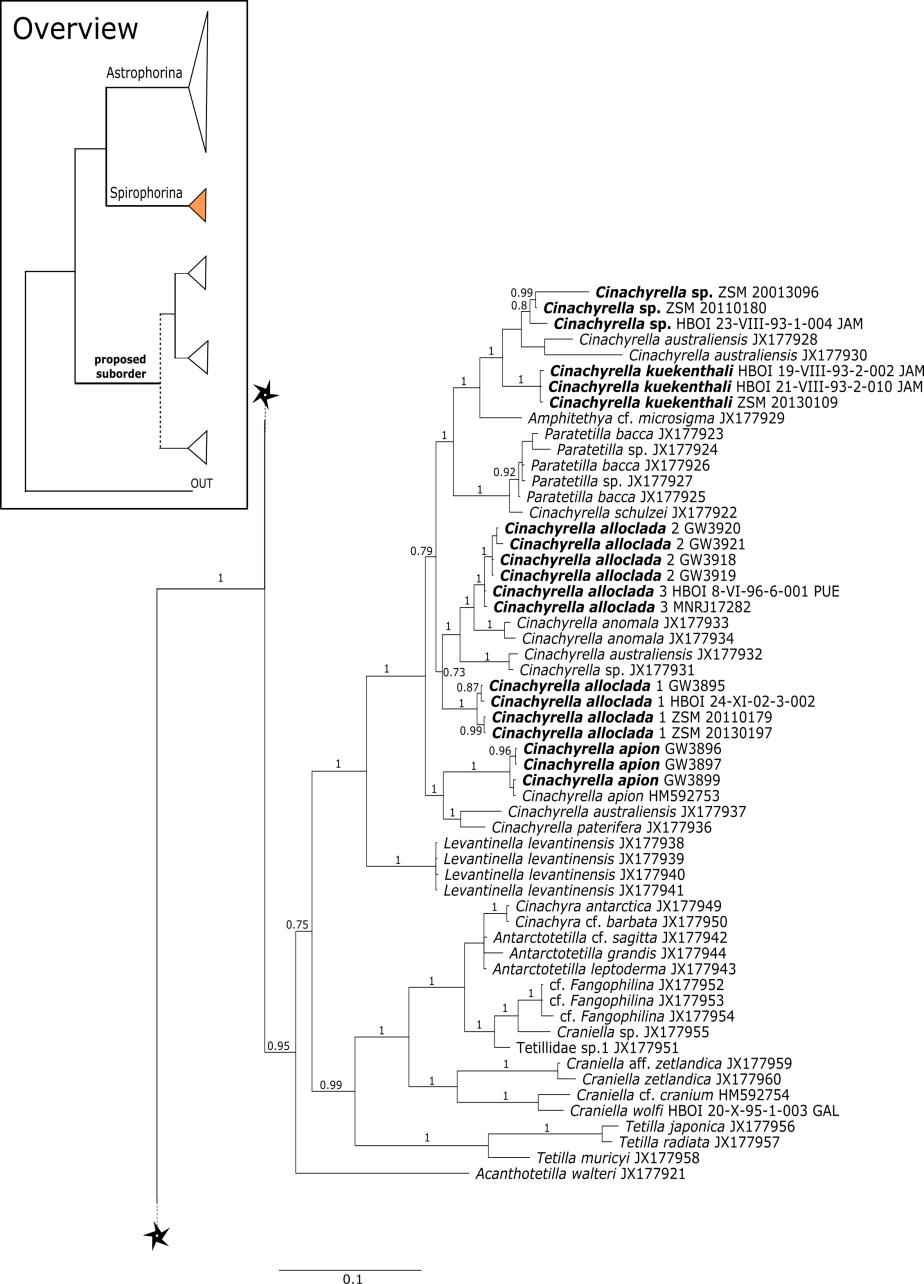
28S phylogeny continued. See caption in Fig. 3.

**Figure 8:**
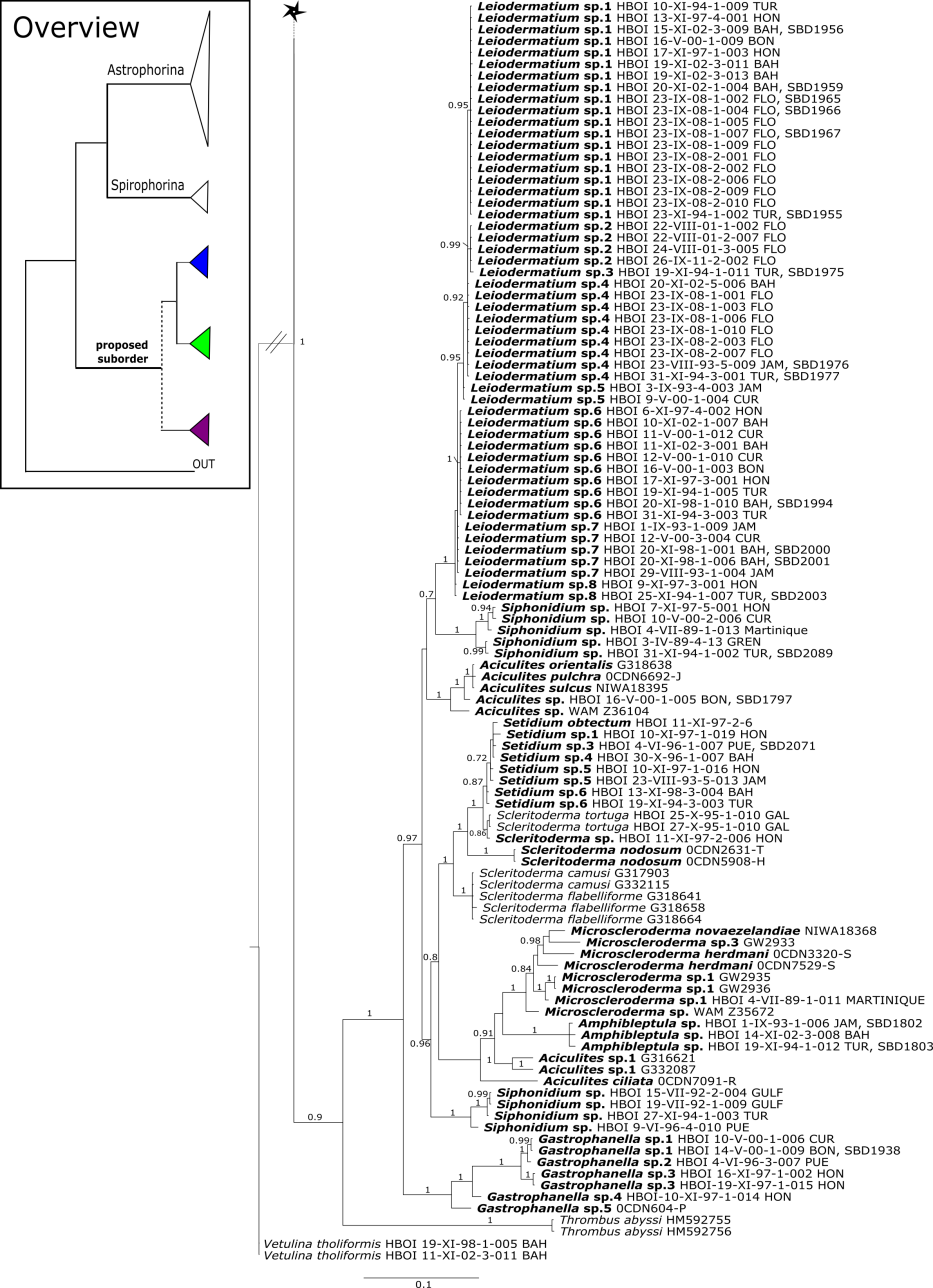
28S phylogeny continued. See caption in Fig. 3.

**Figure 9:**
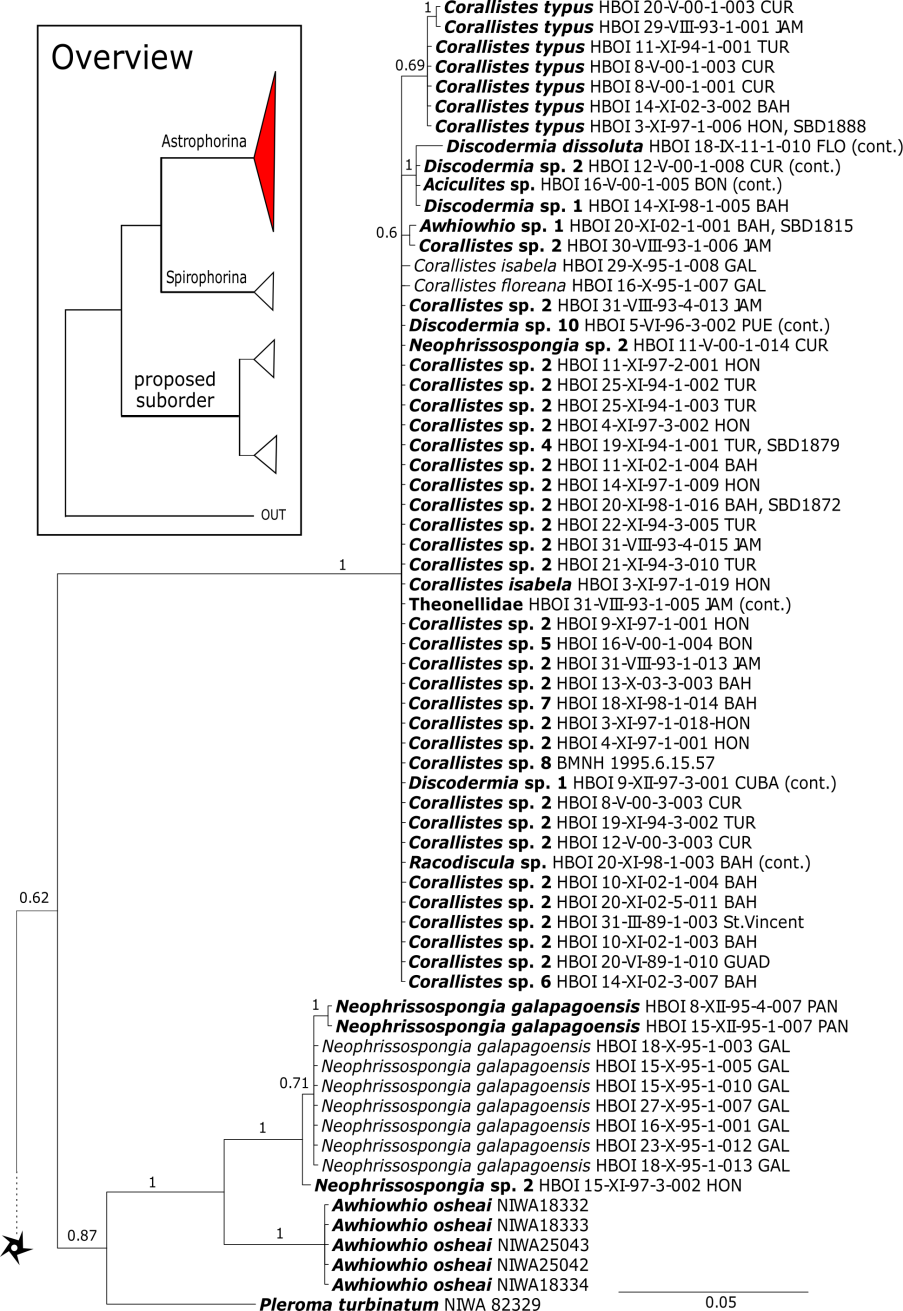
Bayesian Inference phylogeny of Tetractinellida based on *cox1*. Posterior probability (PP) values are provided above or below branches. Self-generated sequences are in bold. Numbers behind taxon names are either voucher numbers or GenBank/ENA accession numbers. Three letter code behind voucher numbers corresponds to the different locations (GULF, Gulf of Mexico; CUR, Curaçao; BON, Bonaire; GUAD, Guadaloupe; PUE, Puerto Rico; JAM, Jamaica; HON, Honduras; TUR, Turks & Caicos; BAH, Bahamas; FLO, Florida; GAL, Galápagos). Taxa where the morphology was investigated are indicated with their corresponding SBD#.

**Figure 10:**
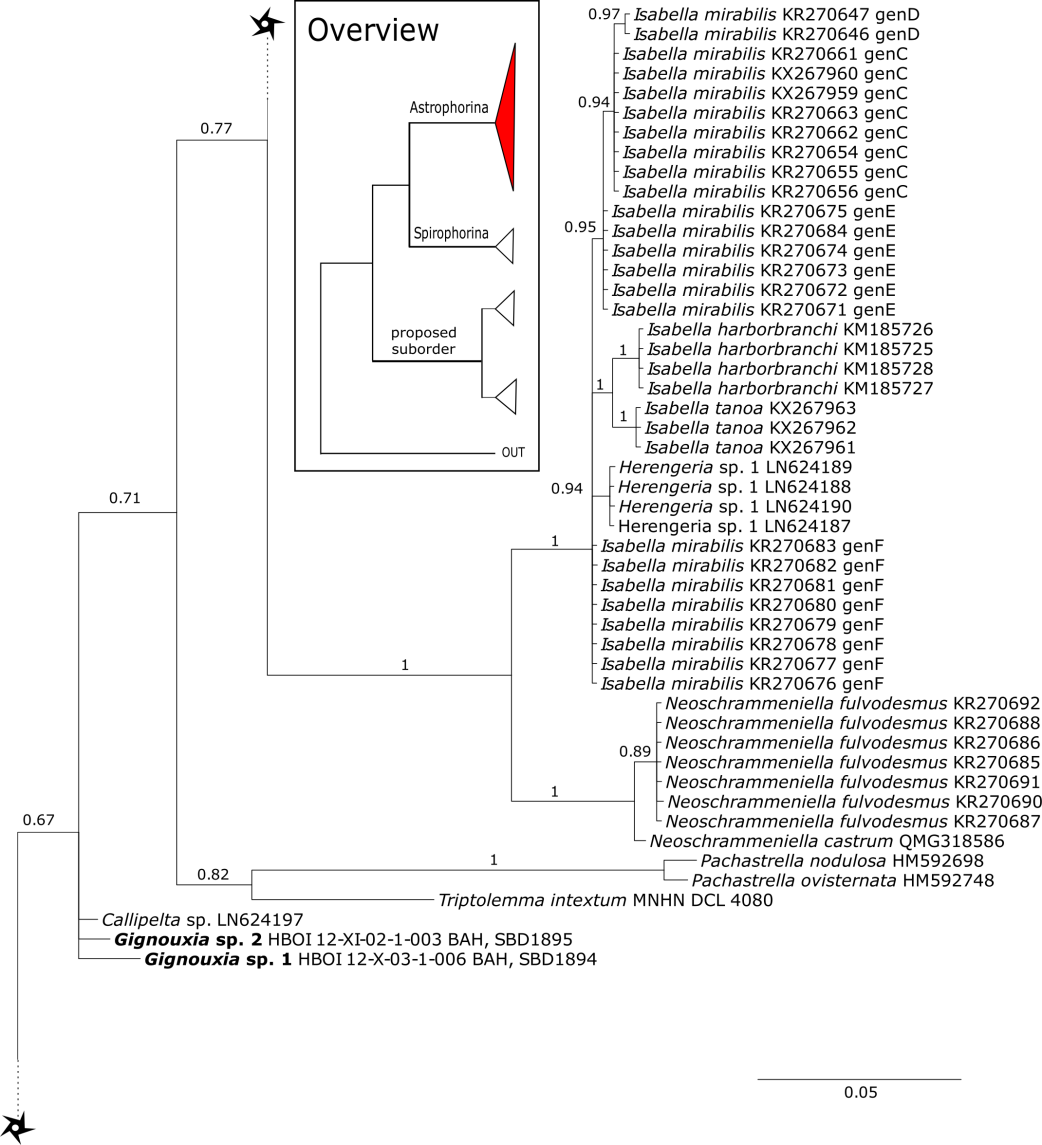
*Cox1* phylogeny continued. See caption in Fig. 9.

**Figure 11:**
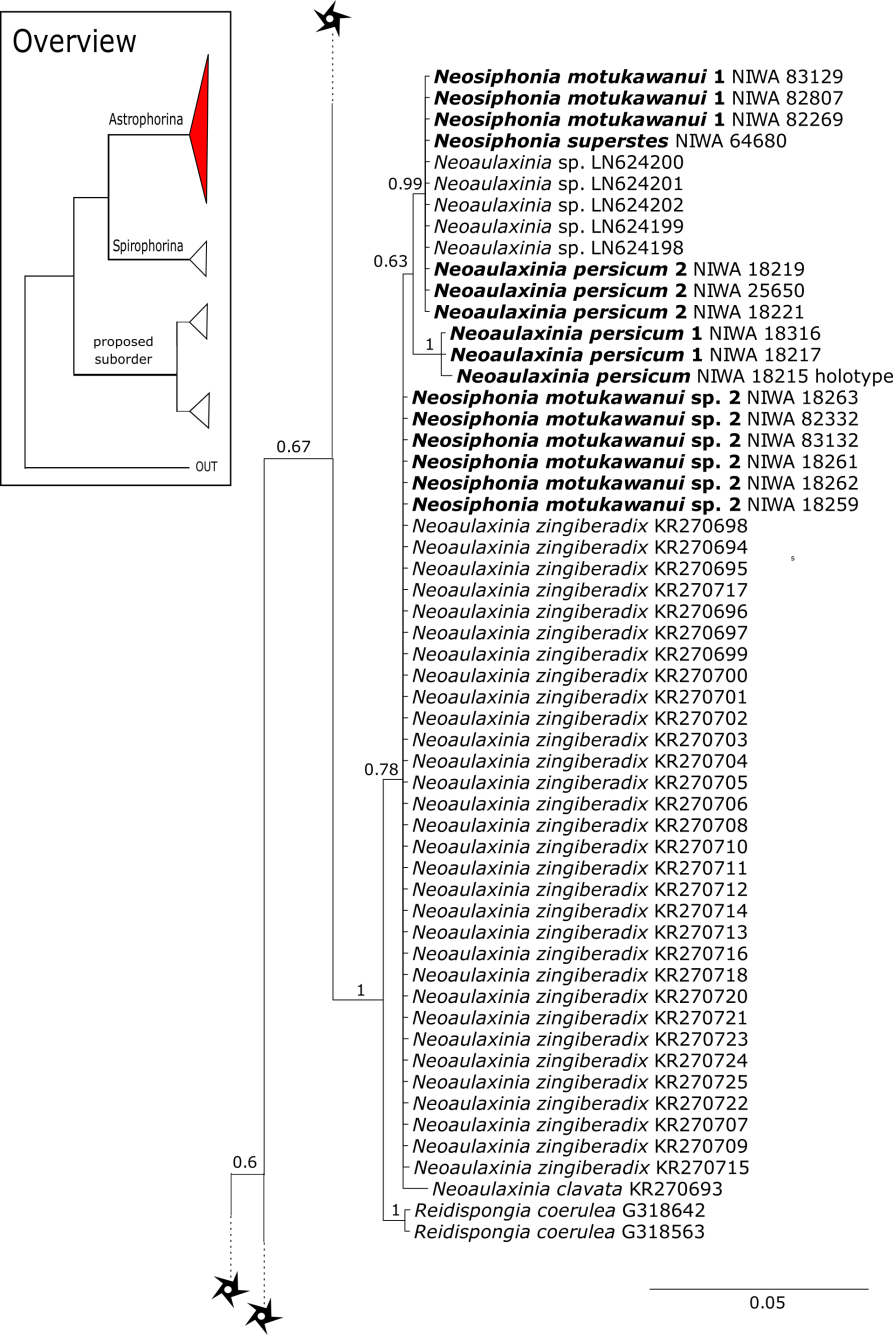
*Cox1* phylogeny continued. See caption in Fig. 9.

**Figure 12:**
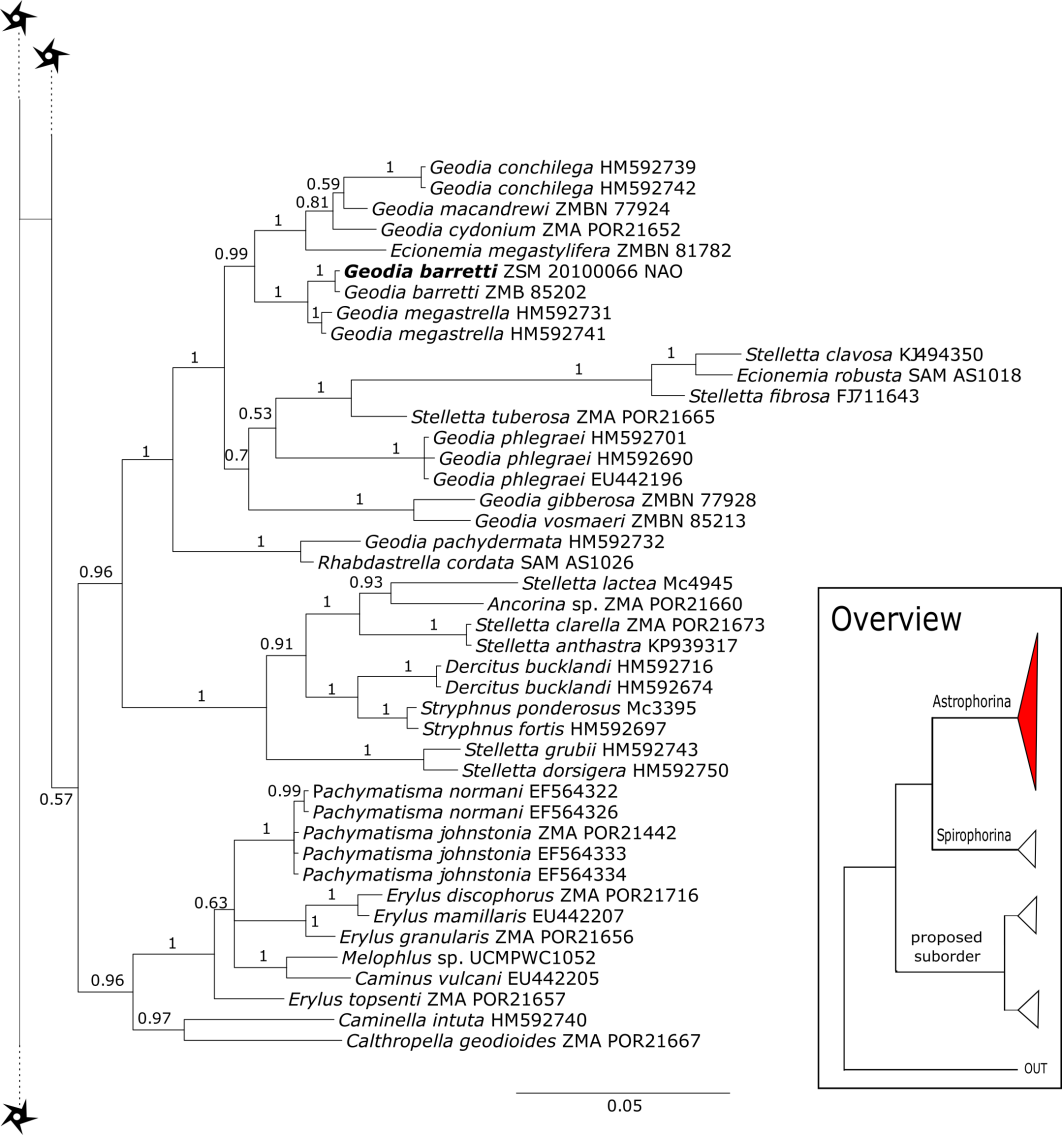
*Cox1* phylogeny continued. See caption in Fig. 9.

**Figure 13:**
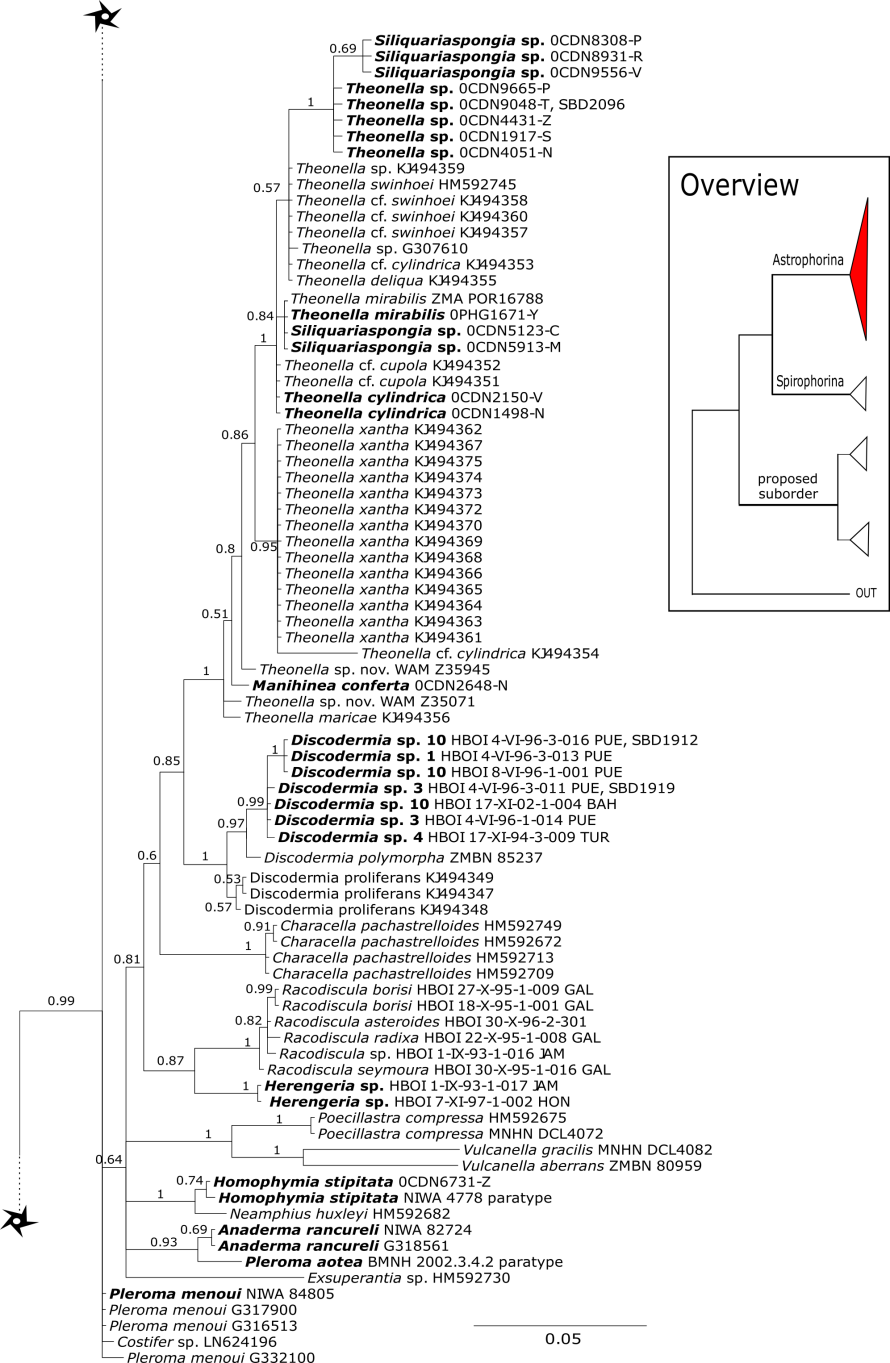
*Cox1* phylogeny continued. See caption in Fig. 9.

**Figure 14:**
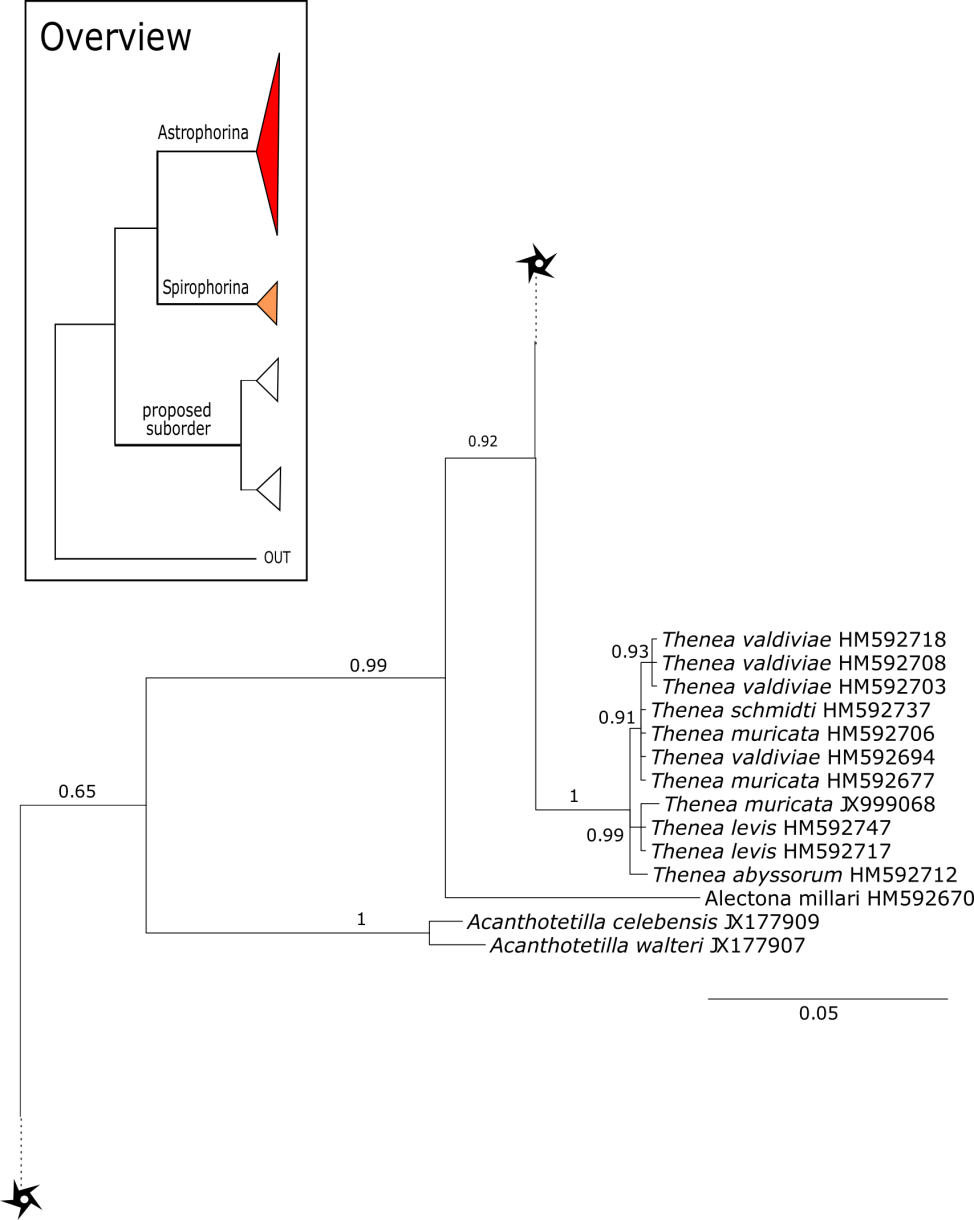
*Cox1* phylogeny continued. See caption in Fig. 9.

**Figure 15:**
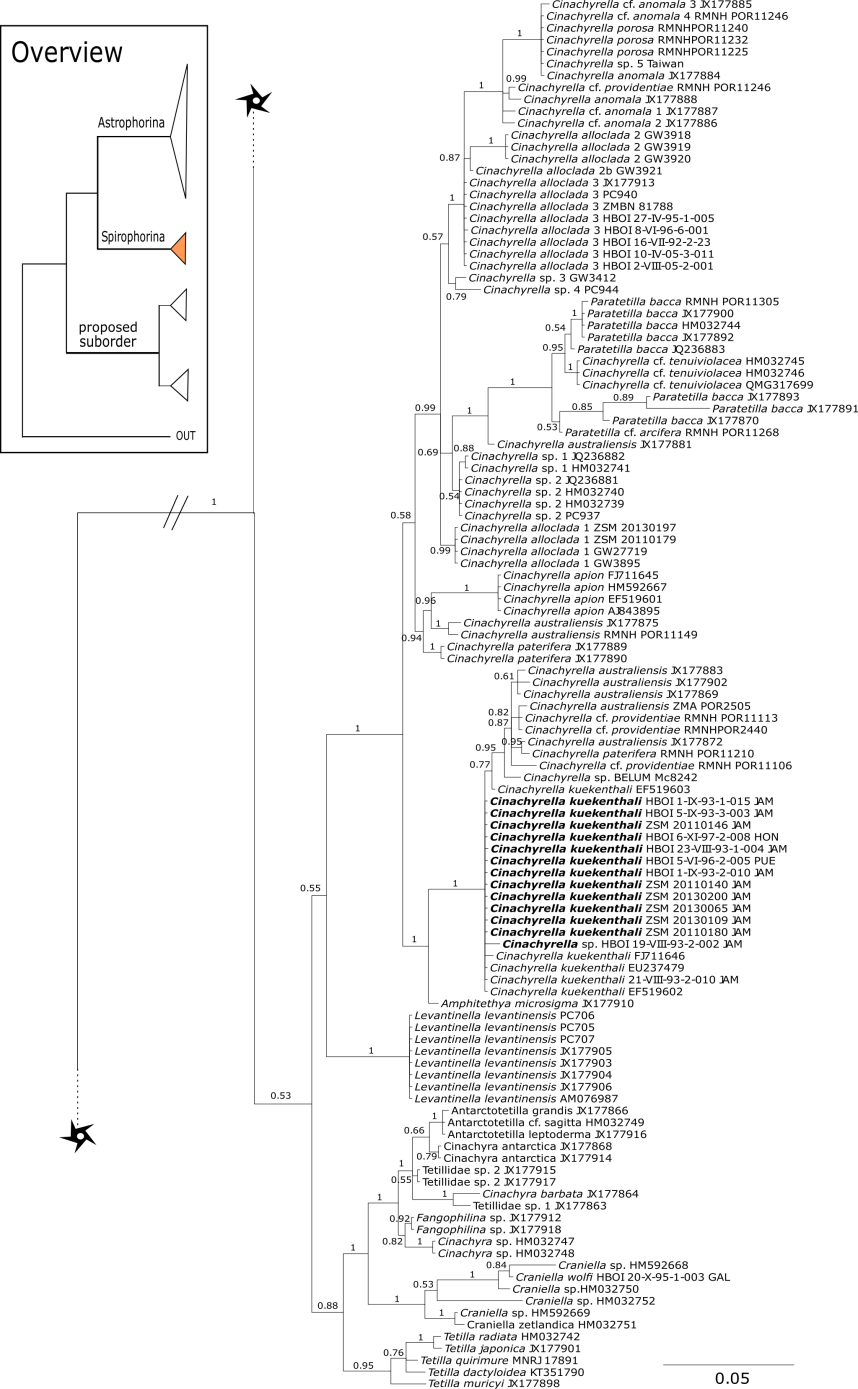
*Cox1* phylogeny continued. See caption in Fig. 9.

**Figure 16:**
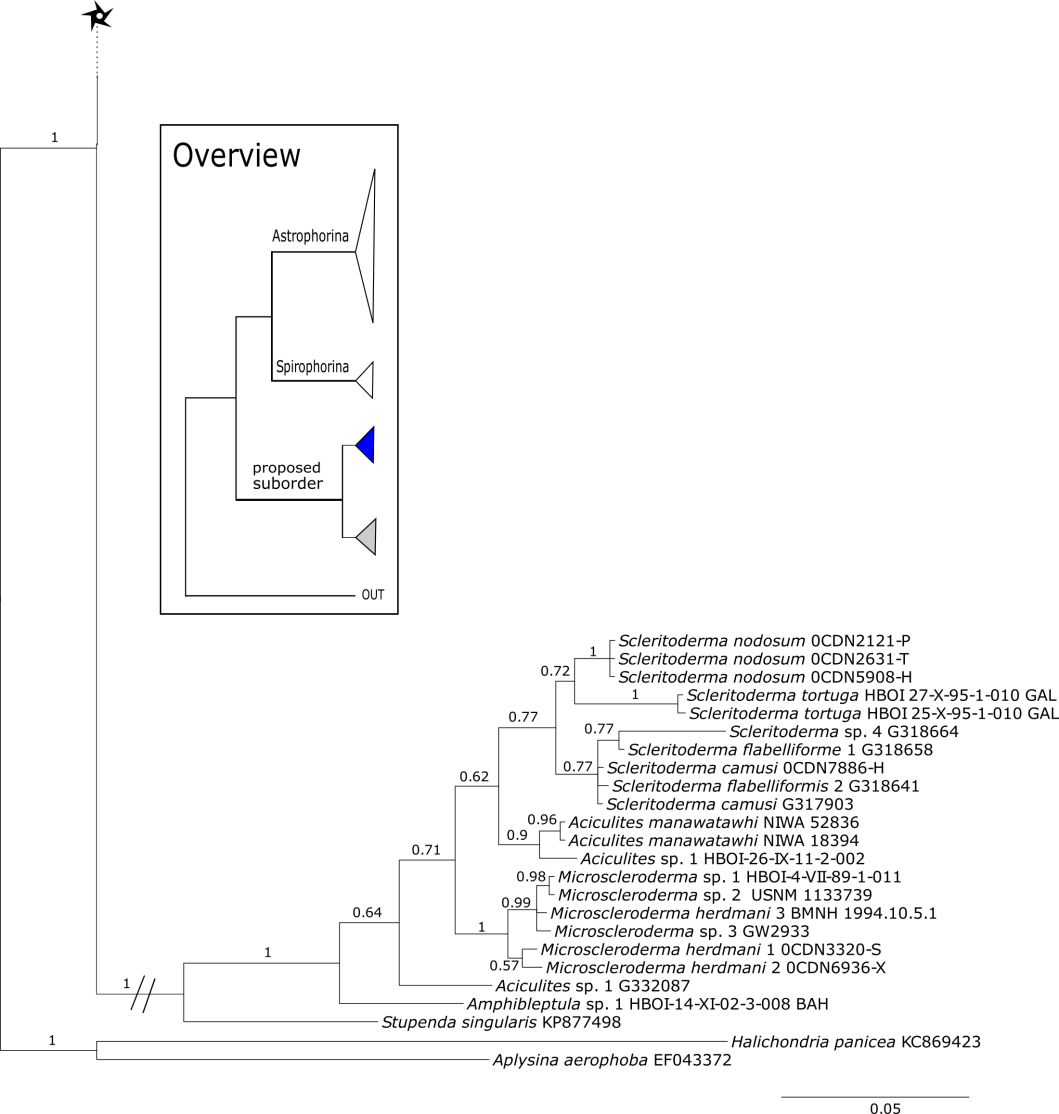
*Cox1* phylogeny continued. See caption in Fig. 9.

The 296 lithistid sequences of at least 88 species from 27 genera (35 known) constitute the largest and most comprehensive taxon set on desma-bearing tetractinellids to date. Our phylogenies (overview in Fig. 2, see Figs. 3–8 (28S) and Figs. 9–16 (*cox1*) for details) corroborate the monophyly of Tetractinellida, currently including the suborders Astrophorina and Spirophorina (Morrow & Cárdenas, 2015) (Fig. 2C). In addition, the affinity of eight desma-bearing families to the suborder Astrophorina (Cárdenas et al., 2011; Morrow & Cárdenas, 2015; Schuster et al., 2015) is confirmed (Figs. 2A–2C). The 28S phylogeny (Fig. 2A) indicates a sister relationship of Astrophorina and Spirophorina. In both gene trees (Figs. 2A–2B) desma-bearing tetractinellids do not group with the Spirophorina (only represented by the Tetillidae in our sampling). In both gene trees the rhizomorine-bearing families Scleritodermidae, Siphonidiidae and Azoricidae form a clade (Fig. 2). However, *Gastrophanella* (Siphonidiidae) is distinct and sister (1.0 Posterior Probability (PP)) to Scleritodermidae/Siphonidiidae/Azoricidae in the 28S phylogeny (Fig. 2A). This sister-group relationship could not be corroborated by *cox1* analysis as no sequence of *Gastrophanella* could be generated. We suspected an intron insertion within *cox1* due to the discovery of these in closely related rhizomorine-bearing genera (*Setidium* Schmidt, 1879, *Microscleroderma* Kirkpatrick, 1903, *Aciculites* Schmidt, 1879, *Scleritoderma* Sollas, 1888) (Schuster et al., 2017). Based on this, various primer sets suggested by Schuster et al. (2017) were tested, however, without success. We suspect that *Gastrophanella* has one or several intron insertions in the *cox1* gene in a yet unknown position. By including several additional rhizomorine-bearing genera such as *Gastrophanella*, *Leiodermatium*, *Siphonidium* and *Amphibleptula* in our datasets, the family Thrombidae could not be recovered within Astrophorina as hypothesized by the Systema Porifera (Hooper & Van Soest, 2002). The 28S gene tree recovers Thrombidae as sister to all rhizomorine-bearing tetractinellids, but this relationship is not supported (0.72 PP) (Figs. 2 and 8) and needs further investigation, including also 28S for Stupendidae Kelly & Cárdenas, 2016, a recently established new family (Kelly & Cárdenas, 2016). In the *cox1* phylogeny (Fig. 2B) Stupendidae is a highly supported sister taxon to Scleritodermidae/Siphonidiidae/Azoricidae. It should be noted that Thrombidae and *Gastrophanella* are missing in the *cox1* phylogeny (Fig. 2B).

#### Intra-subordinal relationships of astrophorine ‘lithistids’

The majority (15 out of 23) of the currently known tetractinellid families are located within the Astrophorina (Morrow & Cárdenas, 2015). This includes eight desma-bearing families (Corallistidae, Isoraphiniidae Schrammen, 1924, Macandrewiidae Schrammen, 1924, Neopeltidae, Pleromidae, Phymaraphiniidae Schrammen, 1924, Phymatellidae and Theonellidae) and seven non-desma bearing families (Cárdenas et al., 2011; Morrow & Cárdenas, 2015; Schuster et al., 2015) (see also Figs. 2A–2C). Thus, the present study supports earlier findings, which were based on lower taxon sampling and additionally provides deeper insights into the intraspecific relationship of desma-bearing astrophorids.

The family **Theonellidae** consists of the genera *Discodermia* Du Bocage, 1869, *Manihinea* Pulitzer-Finali, 1993, *Racodiscula* Zittel, 1878, *Siliquariaspongia* Hoshino, 1981 and *Theonella* Gray, 1868. Theonellidae possesses tetraclone desmas and phyllotriaenes to discotriaenes as characteristic megascleres. Typical microscleres are acanthorhabds, spirasters and amphiasters (Pisera & Lévi, 2002a). Until now, only *Theonella* and *Discodermia* species as well as one *Manihinea* sp. were sequenced in different phylogenetic studies using 18S, 28S and *cox1* (see e.g., Redmond et al., 2013; Hall, Ekins & Hooper, 2014; Schuster et al., 2015). By providing sequences for all known genera, our 28S phylogeny (Fig. 3) recovers Theonellidae as monophyletic (PP = 1.0), thus conclusively support earlier findings of Schuster et al. (2015), while the *cox1* phylogeny (Fig. 13) lacks support in this respect. The 28S phylogeny indicates the monophyly of the genera *Discodermia*, *Manihinea*, *Racodiscula* and a potential new taxon, here denoted as Theonellidae gen. sp., a potential new genus mainly distinct by the layered network of tetraclone desmas with smooth rays and strongly tuberculated tips and the less abundant microscleres on the ectosome (SBD#2102–2106). The sister relationship of *Manihinea conferta* to *Theonella* sp. is highly supported (PP = 0.99) by 28S (Fig. 3), whereas it is not supported by *cox1* (Fig. 13). A close relationship of *Theonella* and *Manihinea* was observed in an earlier study by Redmond et al. (2013) using a nearly complete 18S gene fragment, but unsupported. The genus *Discodermia* is sister to a clade consisting of *Manihinea* +*Theonella* +*Siliquariaspongia*, which is sister to *Racodiscula* +Theonellidae gen. sp.

The genus *Racodiscula* is highly supported (PP=1.0) as sister to Theonellidae gen. sp. Although the outer morphology of Theonellidae gen. sp. (SBD#2106 A–D) is very similar to that of *Racodiscula*, it differs in spicule composition, desma and skeleton structure: the usually abundant spinose microacanthorhabds, covering the surface of *Racodiscula* species (SBD#2065) building a dense crust on the surface, are rarer or even absent in Theonellidae gen. sp. Instead of microacanthorabds, phyllo- to discotriaenes are the main components of the dense surface crust. In addition, Theonellidae gen. sp. possesses desmas with smooth rays and strongly tuberculated tips (SBD#2105 and SBD#2106) building a layered network (SBD#2102), which clearly differs from *Racodiscula* (Schuster et al., 2018).

*Theonella mirabilis* (De Laubenfels, 1954) was first named within the homosclerophorid genus *Placinolopha* (Class Homoscleromorpha, Order Homosclerophorida, Family Plakinidae) on the possession of what de Laubenfels described as ’lophotetractines’. The key size and shape differences between the ’tetralophs’ of *T. mirabilis* and other *Placinolopha* species were noted by Muricy & Diaz (2002), who suggested that the species *mirabilis* had a more likely affinity with species in family Theonellidae. Sequences of 28S (Fig. 3) unite specimens identified as *Theonella mirabilis* in a single clade with a specimen identified as *T. conica* (Kieschnick, 1896) which also has tetraloph-like desmas, suggesting that species with non-articulated ’tetraloph’ desmas may be monophyletic and separate from other *Theonella* spp. However, *cox1* sequences (Fig. 13) separate *T. mirabilis* into two groups, nesting them within diverse species of *Theonella*. *Theonella mirabilis* is very similar in spicule complement to the type species of the genus *Siliquariaspongia*, *S. japonica* Hoshino, 1981 (Family Theonellidae), although the latter lacks the strongyles and possesses frilly discotrianes, the latter occasionally recorded in *T. mirabilis*. Our phylogenies clearly place all of the sequenced *Theonella mirabilis* species within the *Theonella* +*Manihinea* clade (Figs. 3 and 13), confirming that this species belongs to the family Theonellidae. This result is supported by the discovery of potent new depsipeptides mirabamides A-D, that inhibit HIV-1 infection, adding to a small class first exemplified by the papuamides from various *Theonella* spp. (Plaza et al., 2007).

The family **Macandrewiidae** is monogeneric with currently seven valid species (Van Soest et al., 2018a). Until know, only *Macandrewia rigida* Lévi & Lévi, 1989 from the Solomon Islands has been sequenced (28S C1-D2 region, LN624160, G317931) (Schuster et al., 2015). The present study includes a further sequence of an undescribed *Macandrewia* sp. from the Bahamas (909 m depth), which clearly differs from *M. rigida* (Fig. 4). Morphological differences in desmas (SBD#2004) corroborate the genetic difference to *M. rigida* and provide further evidence of a possible new species, which would be the first record in the TWA. Nevertheless, further morphological observations and comparison with the type material of *M. rigida* as well as its sequences are needed to conclusively describe and distinguish this potential new species. Both *Macandrewia* species group within the Geodiidae, close to the Erylinae Sollas, 1888, within a clade of non-desma bearing astrophorins (*Calthropella* Sollas, 1888, *Caminella* Von Lendenfeld, 1894) (Fig. 4). This relationship is currently not supported by morphology (Cárdenas et al., 2018) and in any case suggests a distinct evolutionary history of *Macandrewia* to other ‘lithistid’ families Corallistidae and Neopeltidae (Schuster et al., 2015), where *Macandrewia* was previously allocated (Kelly, 2000; Pomponi et al., 2001).

The family **Phymatellidae** currently includes three genera: *Neoaulaxinia*, *Neosiphonia* and *Reidispongia*. A highly supported sister group relationship of *Neoaulaxinia* Pisera & Lévi, 2002e to the genus *Gignouxia* Moret, 1926 is observed and *Reidispongia coerulea* Lévi & Lévi, 1988 is sister to this clade (Fig. 4). Morphological characters of both sequenced *Gignouxia* species were further investigated and illustrated (SBD#1894). Both species are potentially new to science. *Gignouxia* sp. 1 (SBD#1894) possesses the characteristic pseudophyllotriaenes (SBD#1894) known from the Neopeltidae, while *Gignouxia* sp. 2 (SBD#1895) possesses dichotriaenes characteristic for the Phymatellidae. These dichotriaenes, however, have a unique shape with indented cladomes (SBD#1895). A spicule drawing of *Corallistes tubulatus* Van Soest & Stentoft, 1988 from Barbados, now *Neophrissospongia tubulata*, resembles those unique dichotriaenes implying that *N. tubulata* may need to be reallocated to *Gignouxia*. Interestingly, dichotriaenes of *Gignouxia* sp. 2 resemble those of the fossil *Gignouxia niciensis* Moret, 1926 (Corallistidae) from the Late Cretaceous (Pl. XVIII, Fig. 2, 2′fig-txt 37). We suggest to allocate *Gignouxia* to the family Phymatellidae. *Gignouxia* will include *Gignouxia* sp. 2 as well as *N. tubulata* comb. nov.

**Neopeltidae** polyphyly is given by the highly supported (PP = 1.0) sister relationship of the newly sequenced species *Neopelta* sp. to the non-desma bearing astrophorid *Neamphius huxley* Sollas, 1888 (Fig. 4). Morphologically, these two species only share choanosomal amphiasters with spiny rays and microxeas. Thus, monocrepid desmas and pseudodiscotriaenes characterizing *Neopelta* were lost in *Neamphius*. Spicule losses and gains are not uncommon within tetractinellids and have frequently been shown (Chombard, Boury-Esnault & Tillier, 1998; Cárdenas et al., 2011; Schuster et al., 2015). Nevertheless, these two genera form a robust sister clade to a non-desma bearing Ancorinidae clade consisting of *Stelletta* Schmidt, 1862, *Ancorina* Schmidt, 1862, *Asteropus* Sollas, 1888 and *Stryphnus* Sollas, 1886 (see Fig. 4).

**Corallistidae** is another major family dominating the HBOI collection and subsequently our phylogenies (Figs. 5 and 9). These taxa were the focus of biomedical investigations (e.g., Haar et al., 1996; Wright, 2010), thus targeted during the HBOI expeditions. Even though *Corallistes* were frequently sampled in the past and 15 species are described to date (Van Soest et al., 2018b), only five sequences are published (Kelly Borges & Pomponi, 1994; Chombard, Boury-Esnault & Tillier, 1998; McInerney, Adams & Kelly, 1999). With 52 *Corallistes* specimens sequenced, this study presents the largest data set to date and reveals the monophyly of this genus (28S, PP = 1.0, Fig. 5). *Corallistes typus* (Schmidt, 1870) specimens, type species of the genus, were examined (SBD#1888, 1889, 1892) and sequenced (Figs. 5 and 9). In addition, a *Corallistes isabela* Desqueyroux-Faúndez & Van Soest, 1997 sample from Honduras was sequenced. Until this study *Corallistes isabela* was only known from the Eastern Pacific (Galápagos) and discussed as endemic to the Galápagos (Desqueyroux-Faúndez & Van Soest, 1997 and Schuster et al., 2018). In addition, six *Corallistes* (*C.* sp. 2 to *C.* sp. 7, see Fig. 5) differ by 1–3 bp in the 28S fragment, while no differences were found in *cox1* (Fig. 9). Morphological differences are observed between *C.* sp. 2 (SBD#1872) and *C.* sp. 4 (SBD#1879). For example, *C.* sp. 2 has long (700 µm) thin ectosomal oxeas (SBD#1872 A), while *C.* sp. 4 has ectosomal styles (SBD#1879 D) and subectosomal microxea with spined surfaces (SBD#1879 C). Morphological identifications are in progress and necessary to discriminate the remaining *Corallistes* species.

The polyphyletic corallistid genus *Herengeria* Lévi & Lévi, 1988, only known from the Pacific Norfolk Ridge, New Caledonia, and New Zealand (Schlacher-Hoenlinger, Pisera & Hooper, 2005; Kelly et al., 2009), was sequenced (Fig. 5) and morphologically illustrated (SBD#1949) in the present study for the first time from the Bahamas and Honduras, representing a new genus for the Atlantic. Only two species of *Herengeria* are described (*H. auriculata* Lévi & Lévi, 1988) and *H. vasiformis* Schlacher-Hoenlinger, Pisera and Hooper, 2005). The morphological observations delimit the new TWA species from *H. auriculata* (Schlacher-Hoenlinger, Pisera & Hooper, 2005) due to a lack of subectosomal rhabd-like spirasters. With respect to molecular markers, *Herengeria* spp. from the TWA are distinct from *H. vasiformis*/*Herengeria* sp. from the Pacific (Fig. 5). Our 28S (Fig. 5) and *cox1* (Fig. 10) phylogenies strongly support *Neoschrammeniella* Pisera & Lévi, 2002d as sister to the *Herengeria*/*Isabella* clade. Currently, seven valid species of *Neophrissospongia* (Pisera & Lévi, 2002d) are described (Van Soest et al., 2018c). Only a few *Neophrissospongia* sequences (two 28S and one 18S) from the Pacific Ocean are currently published. However, in order to gain a better understanding of their geographical distribution and genetic differences, additional material from the Caribbean were sequenced in this study: Their resulting 28S phylogeny clearly separates *Neophrissospongia* from the Pacific and the Caribbean Islands. *Neophrissospongia* sp. 1 from different Caribbean Islands is sister (PP=1.0) to *N. microstylifera* and *Neophrissospongia* sp. 3, from the Pacific (Fig. 6). A further Caribbean *Neophrissospongia* species (sp. 2) forms a robust sister clade to *Neophrissospongia* from the Eastern Pacific (Galápagos, Panama) (Fig. 6).

A clade of six as yet unidentified specimens from the HBOI collection (SBD#1814) is sister to the monophyletic *Neophrissospongia* (PP = 1.0, Fig. 6). We assume that this clade consists of species from the as yet unsequenced genus *Awhiowhio* Kelly, 2007 from the Pacific based on morphological evidence. These show similar mega- and microsclere types to *Awhiowhio* such as dicranoclone desmas and smooth dichotriaenes in *Awhiowhio* sp. 1 from the Bahamas (SBD#1815), most similar to the *Awhiowhio osheai* from New Zealand (Kelly, 2007), but slightly different in terms of desma ornamentation. Streptaster microscleres and acanthose microrhabds in *Awhiowhio* sp. 1 (SBD#1814, 1815) differ from those in *Awhiowhio osheai* in sizes and shapes (SBD#1814). The *cox1* phylogeny (Fig. 9) indicates the sister group relationship of *Awhiowhio osheai* Kelly, 2007 to *Neophrissospongia*. A close relationship of *Awhiowhio* to *Herengeria* as suggested by Kelly (2007) based on morphological features is not supported by any of our phylogenies. Instead, both markers independently suggest a close relationship (strongly supported by PP = 1.0) to *Neophrissospongia*. The genus *Pleroma* Sollas, 1888 (family Pleromidae) is recovered as paraphyletic in both phylogenies (Figs. 5, 6, 9 and 13). *Pleroma menoui* (Sollas, 1888) is distant to other *Pleroma* spp. (including the type species *P. turbinatum*) in a close relationship to Corallistidae (Figs. 5 and 9).

#### Intra-subordinal relationships of spirophorine ‘lithistids’

The suborder Spirophorina is characterized by sigmaspire microscleres and its members share triaene spicules with Astrophorina. Currently, three families are known: Samidae Sollas, 1888, Spirasigmidae Hallmann, 1912 and Tetillidae Sollas, 1886, whereas the latter is the largest in terms of genera and species (e.g., Van Soest & Hooper, 2002). The relationships of major clades within our *cox1* and 28S phylogenies (Figs. 3–16) were in concordance with the findings of Carella et al. (2016) and Schuster et al. (2017). The latest revised classification of Morrow & Cárdenas (2015) included the desma-bearing families Azoricidae, Scleritodermidae and Siphonidiidae within Spirophorina. Since then, several studies (Schuster et al., 2015; Schuster et al., 2017; Kelly & Cárdenas, 2016) using *cox1*, 18S and 28S markers showed the separation of all rhizomorine-bearing sponges from Spirophorina (=Tetillidae). The present enlarged dataset corroborates again the absence of desma-bearing sponges in Spirophorina and their grouping in a well supported clade along with the Stupendidae and the Thrombidae (Fig. 2). In order to establish this clade as a new taxa, we await further molecular data from the latter two families (work in progress, MK and PC).

#### Subordinal structure of Tetractinellida

Kelly & Cárdenas (2016) provided strong support for families Azoricidae, Scleritodermidae and Siphonidiidae within a proposed suborder, supported in part by the common possession of rhizomorine desmas.

Regarding **Azoricidae**, Maldonado et al. (2015) discovered a dense and large aggregation of *Leiodermatium pfeifferae* Carter, 1873 on seamounts in the Mediterranean building complex reef-forming structures. Even though *Leiodermatium* Schmidt, 1870 has very few diagnostic characters (no microscleres) to discriminate between species (Pisera, 2002; Pisera & Lévi, 2002b), 11 species are valid to date (WPD access Jan. 2020). In the present study we sequenced the 28S C1-D2 fragment for 52 *Leiodermatium* specimens from several regions in the TWA (Fig. 8) representing at least 8 species and the largest sequenced dataset for this genus to date. The monophyly of *Leiodermatium* is highly supported by our 28S phylogeny (PP = 1.0; Fig. 8). The amplification of *cox1* unfortunately failed, most likely due to the presence of introns similar to other rhizomorine-bearing genera like e.g., *Microscleroderma* and *Scleritoderma* (Schuster et al., 2017). Preliminary morphological investigations (SBD# 1966, 1967, 1959, 1965, 1956, 1955, 1975, 1977, 1976, 1994, 2000, 2001, 2003) adumbrate detailed differences of *Leiodermatium* spp., in particular their surfaces (oscules and ostia sizes), diactines and desma morphology. For instance *Leiodermatium* sp. 1 (SBD#1955) has large and marginate oscules, while *Leiodermatium* sp. 6 (SBD#1994) and *Leiodermatium* sp. 8 (SBD#2003) have large but elevated oscules on exterior margins, in contrast to *Leiodermatium* sp. 7, whose oscules are small and closely distributed. Based on molecular and morphological data we propose eight different species of *Leiodermatium* (Fig. 8) in the TWA, however further morphological investigations are needed to corroborate this assumption. *Leiodermatium* is unsupported (PP = 0.65) sister to a clade of *Siphonidium* spp. (Siphonidiidae); the same relationship was revealed with small fragment of the 18S gene (482 bp) for *Leiodermatium* sp. (Kelly Borges & Pomponi, 1994; Kelly & Cárdenas, 2016). Further investigation and a review of all extant and fossil *Leiodermatium* species is suggested to better understand the geographical distribution and recent diversification of this paleontological important group.

Within the polyphyletic rhizomorine family **Scleritodermidae**, its genera *Aciculites* is polyphyletic and *Scleritoderma* is paraphyletic, while *Microscleroderma*, *Amphibleptula* Schmidt, 1879 and *Setidium* Schmidt, 1879 are monophyletic (Fig. 8). The genus *Amphibleptula* is currently monospecific with *A. madrepora* Schmidt, 1879 from the Caribbean (Pisera & Lévi, 2002c). Morphologically, *A. madrepora* is very similar and easy to confuse with *Microscleroderma spirophora* Lévi, 1960 as discussed in Van Soest & Stentoft (1988). *Amphibleptula* is here sequenced for the first time and our 28S phylogeny shows *Microscleroderma* and *Amphibleptula* sp. 1 as sister groups, although unsupported in the 28S phylogeny (PP = 0.63). Morphological observations (SBD#1802,1803) provide conclusive evidence that our three samples are *Amphibleptula* species, due to their dense tuberculated/blunt spinose rhizoclones, the protruding bundles of oxeas in the oscula area (SBD#1802,1803) as well as the presence of sigmaspires (SBD#1802). Differences to *A. madrepora* are the diactine spicules present in all three *Amphibleptula* sp. 1. In addition, fusiform spined microxeas and acanthorhabds are found in the specimen from Jamaica (HBOI 1-IX-93-1-006, SBD#1802). To conclude, we sequenced two potential new species of *Amphibleptula* with clear unique morphological characters, different from *A. madrepora*.

**Figure 17:**
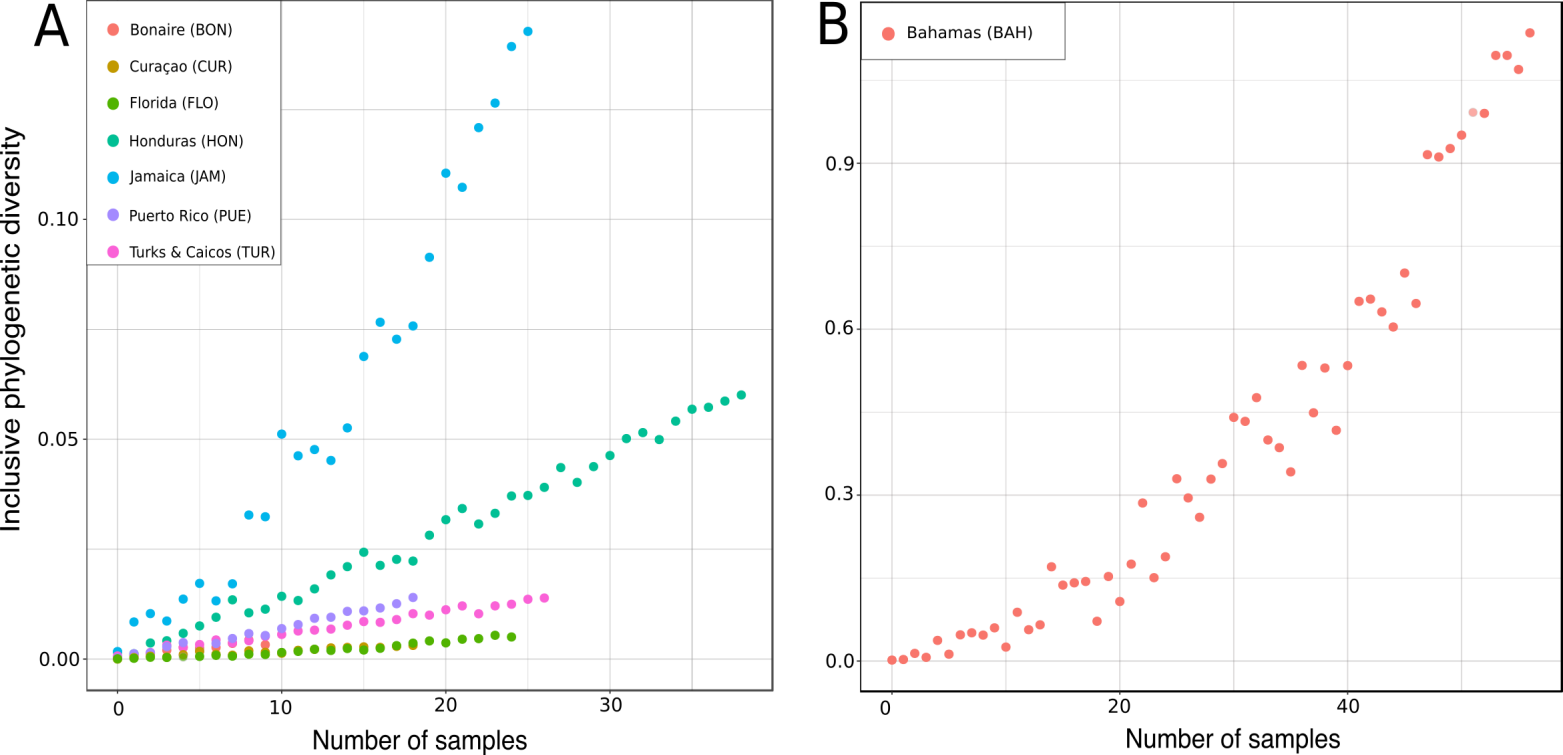
Rarified inclusive phylogenetic diversity (PD_I_) curves per marine regions analyzed. For a better visualization the PD_I_ for the Bahamas are illustrated separately (B) due to their larger number of samples.

**Figure 18:**
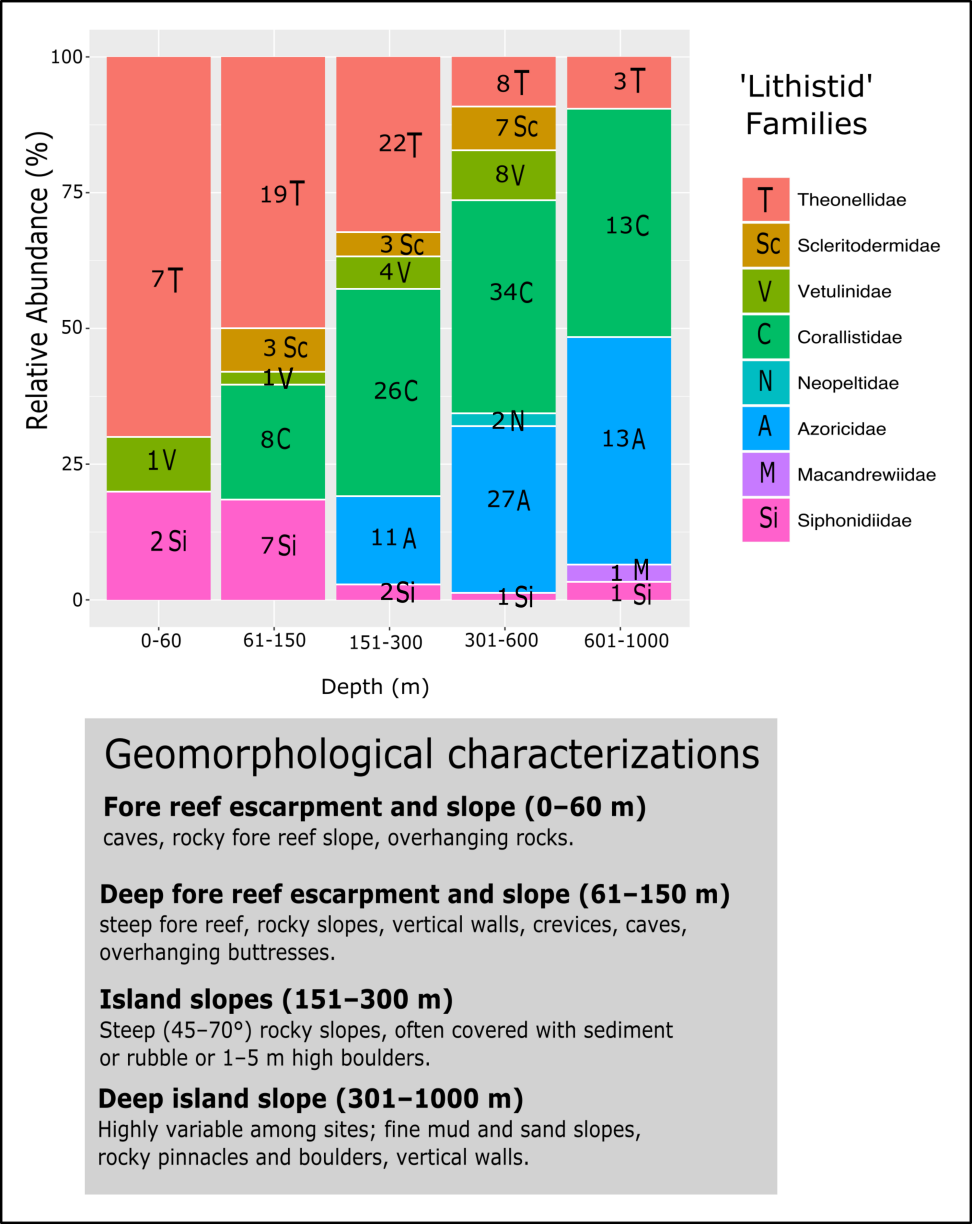
Bathymetric distribution and relative abundance (%) of TWA desma-bearing demosponges based on 234 samples of eight families. Numbers in each bar represent the number of samples investigated. The following genera for each family were included: *Leiodermatium* (Azoricidae); *Corallistes*, *Herengeria*, *Neophrissospongia* and *Awhiowhio* (Corallistidae); *Macandrewia* (Macandrewiidae); *Daedalopelta* and *Neopelta* (Neopeltidae); *Aciculites*, *Amphibleptula*, *Microscleroderma*, *Scleritoderma* and *Setidium* (Scleritodermidae); *Gastrophanella* and *Siphonidium* (Siphonidiidae); *Discodermia*, *Racodiscula* and *Theonella* (Theonellidae); *Vetulina* (Vetulinidae). Geomorphological characterizations of depth zones are given below the graph and follows Pomponi et al. (2001) and Reed & Pomponi (1997).

### Molecular phylodiversity of TWA desma-bearing demosponges

In the present study the inclusive phylodiversity was calculated for Bonaire, Curaçao, Florida, Honduras, Jamaica, Puerto Rico, Turks and Caicos and the Bahamas (Fig. 17). The PD_I_ analyses disclosed a high variation within the TWA locations (Fig. 17). At comparable sampling efforts, the highest PD_I_ was observed in Jamaica (Fig. 17A) indicating a high biodiversity in this region, closely followed by the Bahamas (Fig. 17B). At sample size of 20, Curaçao and Florida showed the lowest PD_I_, followed by Turks and Caicos and Honduras. The high PD_I_ of ‘lithistid’ demosponges calculated for the Bahamas is in agreement with the findings of Reed & Pomponi (1997), and may be explained by the high habitat diversity observed in this region (Reed & Pomponi, 1997) and their close proximity to the species-rich Atlantic (see e.g., Carvalho, Pomponi & Xavier, 2015). Even though Turks and Caicos are close to the Bahamas and the Atlantic, a much lower PD_I_ was calculated, maybe due to less habitat diversity.

### Bathymetric distribution and relative abundance of TWA desma-bearing families

The evaluation of the relative abundance of eight ‘lithistid’ families within each depth zone is based upon 234 specimens collected from eight localities in the TWA (Fig. 18). Theonellidae and Corallistidae are the two dominant families in the present dataset, and assumed to be the dominant families in the TWA (Pomponi et al., 2001). While Theonellidae dominate depth zones of 0–151 m, Corallistidae are more abundant in depth zones of 151–600 m. This corroborates the result of Pomponi et al. (2001) showing that *Discodermia* (Theonellidae) is the dominant genus from 0–151 m, while *Corallistes* (Corallistidae) dominates the zone of 151–914 m. An explanation for this might be that Corallistidae have a dense rigid skeleton of dicranoclone desmas, while Theonellidae possess a less articulated skeleton of tetraclone desmas. Thus, it can be hypothesized, that ‘lithistids’ with a hyper-silicified dense desma skeleton like Corallistidae occur in deeper zones ≥300 m, while those with a less dense desma skeleton like most of the *Theonella* species (Theonellidae) moved into more shallow water habitats, where less silica is available to build their skeleton (e.g., Tréguer et al., 1995). This trend was also observed in the South West Pacific (Kelly et al., 2007; Hall, Ekins & Hooper, 2014). As Corallistidae and Theonellidae are considered to be polymorphic (Pisera & Lévi, 2002a, Pisera & Lévi, 2002d), it is difficult, to draw any conclusion of different depth zones or habitats influencing growth form patterns in these two families.

However, further ‘lithistid’ families with a similar bathymetric trend are observed and growth forms are suggested to play a role in the bathymetric distributions of ‘lithistids’. For instance *Leiodermatium* spp. (Azoricidae) are abundant (27 specimens) in depth zones 301–1,000 m. Similar to Corallistidae *Leiodermatium* possess a dense heavily articulated skeleton, but of strongly spinose rhizoclone desmas. The growth form of *Leiodermatium* species are described as being foliated or vase to ear-shaped (Pisera & Lévi, 2002b). Such growth forms are suggested to improve the water circulation in sponges, in particular of those in the deep-sea habitats, and to be more resistant to higher water viscosity and scarcity of particles (Levinton, 1982; Gage & Tyler, 1991). Many vase to cup or ear-shaped sponges have their inhalant pores facing the outer side and exhalant openings on the upper side separating incoming and processed water (Sará & Vacelet, 1973; Pronzato, Bavestrello & Cerrano, 1998), which may reduce any negative effect on filtering due to a sedimentation. This is in contrast to Siphonidiidae, a family represented in this analysis by the genera *Gastrophanella* and *Siphonidium*, which are rather encrusting or irregular cylindrical, thus more abundant in the depth zone of 61–150 m.

**Scleritodermidae** occurred more often on vertical walls in depth 301–600 m, but was also not observed to be a major component of the ‘lithistid’ fauna in the TWA. The greatest number of desma-bearing demosponges were found in depth zone 301–600 m (87 specimens), with Corallistidae as the dominant family (34 specimens) followed by Azoricidae with 27 specimens. Diverse habitats from fine mud and sand slopes to rock pinnacles, boulders and vertical walls in this depth zone (Fig. 18) could be a possible explanation. The families Neopeltidae and Macandrewiidae are rare in our study with only one species discovered at 909 m depth on a vertical wall in the Bahamas (*Macandrewia* sp.), and two *Daedalopelta* spp. collected from the Bahamas at 301–600 m. This corroborates the findings of Pomponi et al. (2001), because they found one species of *Daedalopelta nodosa* Schmidt, 1879 at 452 m in the Bahamas, one *Neopelta perfecta* in 116 m depth from Grenada and one *Macandrewia clavatella* in the southwest coast of Florida. These families and species are also found to be rare in the Southwest Pacific (Lévi, 1991; Kelly, 2000). Besides the tetractinellid ‘lithistid’ sponges, we noted that other desma-bearing sponge lineages, such as family Vetulinidae (Order Sphaerocladina) constitute only a minor component in any depth-zone in the TWA.

Further testing is required to assess whether geomorphological conditions resulting of a variety of complex tectonic interactions (e.g., strike-slip faults, thrust fault, subduction and seafloor spreading in Cayman Trough, see Fig. 1), directly affect diversity and bathymetric distribution of ‘lithistids’ in the TWA (Fig. 18).

## Conclusion

In summary, this is the first integrative approach using molecular and morphological data on TWA ‘lithistid’ demosponges, thus contributing to a better understanding of their phylogenetic affinities, diversity and bathymetric distribution patterns. The present study points to specimens/groups in need of deeper taxonomic investigations and revision, however, additional morphological as well as other independent markers are needed. With recent evidence (Pomponi et al., 2001) that ‘lithistids’ are dominant components among all investigated TWA regions, we suggest a comparable diversity to the Pacific ‘lithistids’ as well as to the Mesozoic fauna. Furthermore, there is a clear shift of lithistids with a rigid and heavily articulated desma towards deeper habitats (Corallistidae and Azoricidae), whereas ‘lithistids’ with a less articulated skeleton tend to occur in more shallow habitats (Theonellidae and Siphoniidae). A major effect causing this shift is the availability of silica in the ocean throughout time. Our robust phylogeny enables relaxed molecular clock analyses in conjunction with the rich fossil record of lithistids to better correlate such shifts to geological/geochemical events in the past.

##  Supplemental Information

10.7717/peerj.10775/supp-1 Supplemental Information 1 Sample list including museum numbers, metadata and accession numbers Collection Numbers refer to the Sponge Barcoding Database (SBD#), Bavarian State Collection for Paleontology and Geology (SNSB-BSPG.GW), National Institute of Water and Atmospheric Research (NIWA#), United States National Cancer Institute shallow water collection programme contracted to the Coral Reef Research Foundation (0CDN#), Harbor Branch Oceanographic Institute (HBOI#), California Academy of Sciences Invertebrate Zoology (CASIZ#), Queensland Museum (QMG#), Natural History Museum (BMNH#), personal collection of Michelle Kelly (MKB#), Western Australian Museum (WAM Z#), and the Zoological State Collection Munich (ZSM#).

## Additional Information and Declarations

### Field Study Permissions

The following information was supplied relating to field study approvals (i.e., approving body and any reference numbers): Permission was granted to Harbor Branch Oceanographic Institute by: the United States National Oceanic and Atmospheric Administration, National Marine Fisheries Service, Dry Tortugas National Park (letters of acknowledgement F/SER25:KM, F/SER23:PE, SER02-130) and the Florida Keys National Marine Sanctuary (permit numbers 2001-049, 2001-043); the Government of the Bahamas, Department of Fisheries; the Government of Honduras, Department of Fisheries (DIGEPESCA-638/97); the Government of Portugal, Parque Natural de Madeira; the Government of Jamaica, Jamaican Ministry of Foreign Affairs (diplomatic note no. 7/703/315); the Government of Bonaire, Bonaire Marine Park, and Netherlands Ministry of Foreign Affairs (note no. VADV-172/00); the Government of Panama (American Embassy Panama telegram no. 5570); the Puerto Rico Department of Natural And Environmental Resources; the Government of St. Vincent; the Government of France; and the Government of Turks and Caicos (American Embassy London telegram no. 12514).

### DNA Deposition

The following information was supplied regarding the deposition of DNA sequences: Novel sequences are archived at the European Nucleotide Archive: LR655885–LR656163 (28S) and LR698845–LR698952 (CO1).

### Data Availability

The following information was supplied regarding data availability: Data is available at the Sponge Barcoding Database: SBD#1794–SBD#2108. Data is available at GenBank: LR655885–LR656163 and LR698845–LR698952.
